# Transcriptomic profiling and gene network analysis revealed regulatory mechanisms of bract development in *Bougainvillea glabra*

**DOI:** 10.1186/s12870-024-05246-7

**Published:** 2024-06-13

**Authors:** Xiangdong Liu, Yaonan Peng, Qinghui Zeng, Yuwan Ma, Jin Liu, Yaqi Huang, Xiaoying Yu, Jun Luo, Yanlin Li, Meng Li, Fuxiang Cao

**Affiliations:** 1 College of Horticulture, Hunan Agricultural University, Changsha, 410128 China; 2 Hunan Applied Technology University, Changde, 415000 China; 3 Engineering Research Center for Horticultural Crop Germplasm Creation and New Variety Breeding, Ministry of Education, Changsha, 410128 China; 4 Hunan Botanical Garden, Changsha, 410128 China; 5 Hunan Mid-Subtropical Quality Plant Breeding and Utilization Engineering Technology Research Center, Changsha, 410128 China; 6 Yuelushan Laboratory, Changsha, 410128 China; 7 College of Life Science and Technology, Central South University of Forestry and Technology, Changsha, 410004 China

**Keywords:** Bract development, *Bougainvillea glabra*, MADS-box, Squamosa promoter binding protein (SBP), Plant hormone signal transduction, Chlorophyll metabolic pathways

## Abstract

**Background:**

Bracts are important for ornamental plants, and their developmental regulation process is complex; however, relatively little research has been conducted on bracts. In this study, physiological, biochemical and morphological changes in *Bougainvillea glabra* leaves, leaf buds and bracts during seven developmental periods were systematically investigated. Moreover, transcriptomic data of *B. glabra* bracts were obtained using PacBio and Illumina sequencing technologies, and key genes regulating their development were screened.

**Results:**

Scanning electron microscopy revealed that the bracts develop via a process involving regression of hairs and a color change from green to white. Transcriptome sequencing revealed 79,130,973 bp of transcript sequences and 45,788 transcripts. Differential gene expression analysis revealed 50 expression patterns across seven developmental periods, with significant variability in transcription factors such as *BgAP1*, *BgFULL*, *BgCMB1, BgSPL16*, *BgSPL8*, *BgDEFA*, *BgEIL1,* and *BgBH305*. KEGG and GO analyses of growth and development showed the involvement of chlorophyll metabolism and hormone-related metabolic pathways. The chlorophyll metabolism genes included *BgPORA*, *BgSGR*, *BgPPH*, *BgPAO* and *BgRCCR*. The growth hormone and abscisic acid signaling pathways involved 44 and 23 homologous genes, and coexpression network analyses revealed that the screened genes *BgAPRR5* and *BgEXLA1* are involved in the regulation of bract development.

**Conclusions:**

These findings improve the understanding of the molecular mechanism of plant bract development and provide important guidance for the molecular regulation and genetic improvement of the growth and development of ornamental plants, mainly ornamental bracts.

**Supplementary Information:**

The online version contains supplementary material available at 10.1186/s12870-024-05246-7.

## Introduction

Bracts are metamorphosed leaves that accompany the inflorescence. In general, any leaf associated with an inflorescence can be defined as a bract [1, 2]. Strictly speaking, the bract is not an inflorescence structure; rather, it can be considered an extension of floral organs. The bract primordia originating from the stem meristem are usually generated at the very early stage of reproductive development [3]. However, a "bract suppression system" has been observed in many, but not all, angiosperm species. This system stops the development of the bract and eventually subsumes the bract primordia into the floral meristem. Therefore, bracts are absent in many model plants, such as *Arabidopsis*, maize (*Zea mays*) and rice (*Oryza sativa*). The bract development system has been suppressed rather than removed in these taller plants. In rice spikes, *SPLs* and *NL1* synergistically regulate the transcriptome and chromatin accessibility after the reproductive transition, as well as the expression of other bract repressor genes, such as *PLA1*, *PLA2*, and *RI. PLA2* and *PLA3* are integrated into common downstream X factors (one or more unknown genes) to regulate bract repression. Bract repression is not only necessary for flower development but also essential for the transition from asexual to reproductive meristems [4]. A study in rice indicated that bract suppression is not only necessary for floral development but also critical for the transition from vegetative to reproductive branching [5]. Although bract suppression is a conserved mechanism in most angiosperm lineages, some plants, such as species belonging to the *Cornaceae*, *Nyssaceae, Nyctaginaceae* and *Araceae* families, exhibit natural bract development.

In many ornamental plants, the bract is an important organ that participates in reproductive events, as well as an ornamental feature [6]. Bracts with ornamental value are called petaloid bracts, formed as a result of the ectopic expression of genes determining petal identity in the leaf. This process is thought to involve developmental signal transduction and the activation or suppression of related genes. Ectopic petalization (the development of petal-like characteristics in nonpetal organs) contributes to the diversity of flower morphology in the process of angiosperm evolution. Bract development appears to occur due to regulation by ABC/ABCDE class genes of the MADS-box family. Sepal formation is determined by the *AGAMOUS-like 6* (*AGL6*) and *Septin* (*SEP*) genes, but few specific studies have been conducted on this subject. The MADS-box gene family belonging to the B-class of the ABC model has been verified to play a key role in ectopic petalization. A MADS-box gene, *AGL6,* was shown to regulate floral organ development and flowering time in *Arabidopsis* [7]. Overexpression of the *AGL6* gene in *Arabidopsis* promoted the growth of petal-like bracts. Similarly, ectopic expression of MADS-box genes was observed in *Cornus officinalis*. Significant upregulation of the *CorPI-B*, *CorPI-A* and *CorAP3* genes was detected in *C. officinalis* bracts during different developmental stages [8, 9]. In *Aristolochia* (Aristolochiaceae, basal angiosperm), the expression of an *AP3-like* gene was detected only during the late stages of petaloid perianth development, and the expression of B-class paralogs was detected in the late stage of Aquilegia petaloid sepal development [10]. The *JAGGED* (*JAG*) gene is the only gene reported to positively regulate bract development in *Arabidopsis*. Overexpression of the *JAG* gene promoted bract development in *Arabidopsis*, and bract development was inhibited in the *APETALA1* (*AP1*) and *JAG* double mutant [11]. A recent study identified a bract-specific gene, DiASR1 (abscisic acid-, stress- and ripening-related protein), from the dove tree (*Davidia involucrata*). Overexpression of the *DiASR1* gene induced the formation of bract-like leaves in *Arabidopsis* [2]. Although there is a large body of literature on bract development, most of the research focused on bract inhibitory systems and was carried out in model plants, whereas the mechanism of natural bract development remains largely unknown. More genes regulating bract development are expected to be explored in naturally bracted species.

Ornamental plant bracts are currently being studied mainly for their pigments. For example, *Bougainvillea,* a woody plant of *Nyctaginaceae* [12] that is rich in pigments such as chalcone-flavonone isomerase 1 (*CHI1*), 4,5-DOPA dioxygenase extradiol (*DOD*) and flavanone 3-hydroxylase (*F3H*), has been shown to be involved in bract color change [13, 14]. However, there is very little research on how bracts develop, and the molecular mechanism underlying the key events of bract development, including organogenesis, chloroplast degeneration, petal identity determination, rapid growth and abscission, needs to be holistically investigated. *Bougainvillea glabra* 'Mrs. Eva White' bracts have significant ornamental value. They are large and thin, making them ideal for studying bract development. To elucidate the developmental mechanisms of bracts in ornamental plants, we conducted a comprehensive analysis of *B. glabra* 'Mrs. Eva White' at the morphological and transcriptomic levels to identify key genes involved in these processes.

## Results

### Morphological and photosynthetic pigment changes during bract development in *B. glabra*

**Fig. 1:**
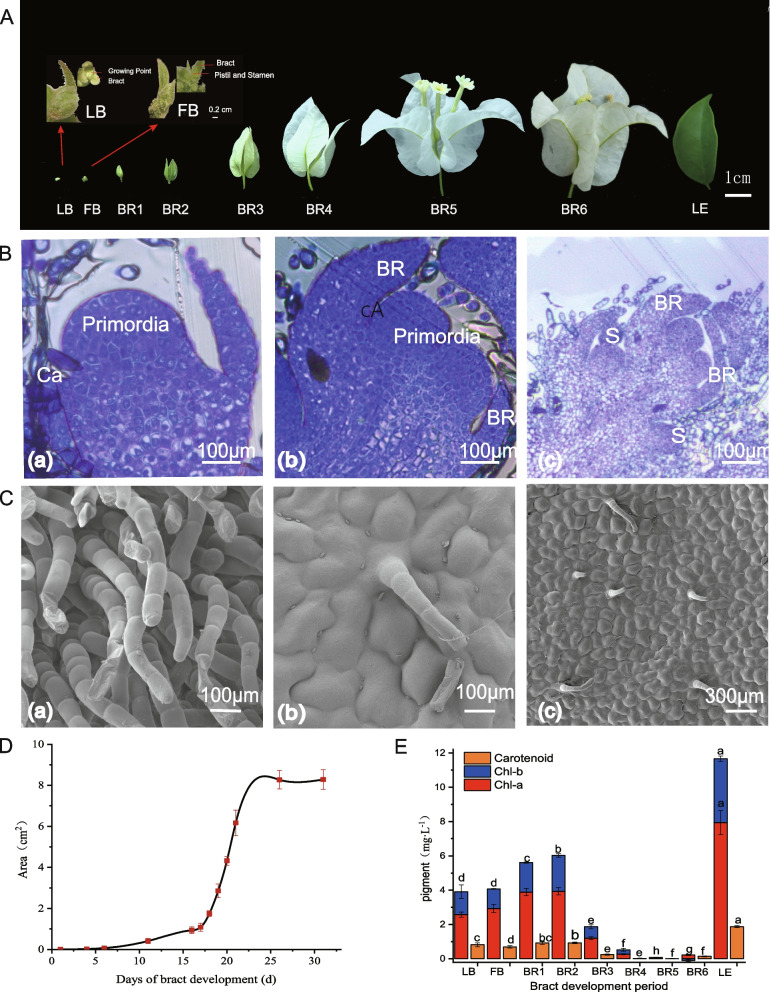
Morphostructural and chlorophyll content changes during bract development in B. glabra. A B. glabra leaf buds, bracts in 7 developmental periods and leaf sampling periods; LB (leaf bud), FB (floral bud), BR (bract), LE (leaf). B Resin section of B. glabra during bud development. **a** Inflorescence primordium differentiation stage. **b** Late stage of floral primordial differentiation. **c** The organs of the third whorl begin to develop consecutively. C Electron microscope image of the bract development process of B. glabra. **a** Electron microscope image of bracts from the BR1 period. **b** Bract fluff shedding. **c** Electron microscopy image of bracts at BR5. D Changes in bract area during bract development in B. glabra. E Chlorophyll and carotene contents of B. glabra leaf buds, bracts and leaves at seven developmental stages

The development of *B. glabra* buds occurs in five main stages, as observed by resin sectioning. At the initial stage, only bracts and growth points are present (Fig. S1A-a). The primordium forms the outermost whorl of the inflorescence, around which the primordium of the bracts appears in rapid succession and consists of outer and inner cells, with the former being larger and looser and the latter being smaller and more compact, followed by gradual growth of the flower primordium (Fig. 1B-a). The floret primordium rapidly differentiates (Fig. S1A-b), and the perianth whorl begins to appear around the primordium (Fig. 1A, S1A-b). As the floral organs develop further, the buds expand, the sepals and petals gradually elongate, the growth cone broadens, the outer petals differentiate into small projections, and the stamen primordia begin to form (Fig. 1B-c). During the LB (late bud) period, the stage of bract protocorm differentiation, the androecium has not yet completed its differentiation and is still in the protocorm stage (Fig. S1B-a). Subsequently, androgynous differentiation is completed, and this stage is known as the stage of floral primordium differentiation (Fig. S1B-b).

Scanning electron microscopy revealed that bract villi gradually degenerated during development (Fig. 1C). During the BR1 period, the villus density was high, and the density gradually decreased as the bracts developed (Fig. S1C). The development of *B. glabra* bracts showed a slow-fast-slow pattern. The bracts were initially green and then showed a change in chlorophyll degradation. From the onset of bract primordium formation, growth was slow for the first 5 days, accelerating on day 6 and entering a period of rapid growth on day 17, when the bracts gradually changed from green to white (Fig. 1D). The chlorophyll content tended to decrease during development, especially significantly after bract period 2, indicating that bract development was accompanied by a process of chlorophyll regreening. However, the chlorophyll content of the bracts increased slightly during senescence. Overall, the chlorophyll and carotene contents of the *B. glabra* bracts were significantly lower than those of the leaf blades (Fig. 1E).

### Transcriptome sequencing and sequence analysis of leaf buds, leaves and bracts at different developmental stages

In this study, the samples were subjected to transcriptome sequencing using the circular consensus sequence technique, which yielded a circular consensus sequence with a total length of 1041,643,869 bp. The homologs and polyA tails were removed by IsoSeq processing, and 463,177 FLNC reads were obtained. Subsequently, the FLNC sequences were clustered and demultiplexed using the ICE tool of SMRTlink software, resulting in a nonredundant transcript sequence of 79,134,466 bp. Thereafter, to improve sequence accuracy, the transcript sequence was further corrected using LoRDEC error correction software, resulting in a corrected transcript sequence of 79,130,973 bp.

After obtaining the transcript sequences, they were clustered and demultiplexed using cd-hit software to create the final full-length transcript sequences, which were used as the reference transcript sequences for second-generation data comparison. A total of 45,788 transcripts were detected in the samples, with a total of 706,647,796 bp and an average length of 1,544 bp. These findings provide an important database for subsequent transcriptome analyses.

### Transcriptomic differential gene expression analysis

**Fig. 2:**
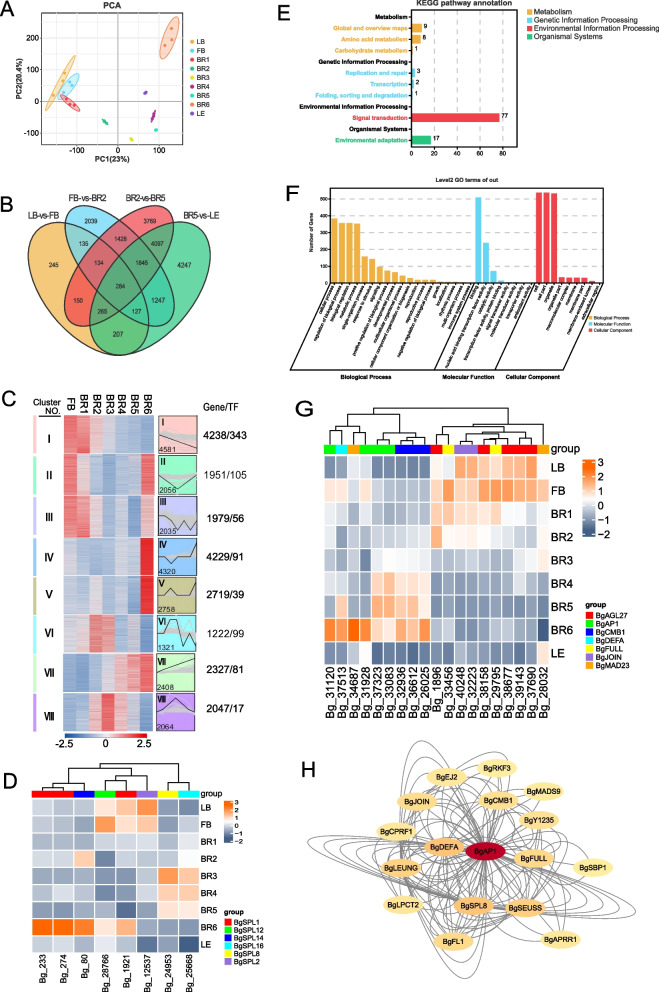
Analysis of genetic differences. **A** PCA plot of the sample FPKM values. **B** Venn diagrams showing differentially expressed genes among the LB/FB, FB/BR2, BR2/BR5, and BR5/LE comparisons. **C** STEM trend analysis plot. **D** KEGG enrichment analysis plot of differentially expressed transcription factors. **E** GO enrichment analysis plot of differentially expressed transcription factors. **F** Heatmap of significant expression trends of genes in the SPL family. **G** Heatmap of differentially expressed MADS-box family members with significant expression trends. H Differential protein interaction network map

Principal component analysis (PCA) of the gene expression levels (FPKM) of all the samples revealed that the samples from the LB, FB and BR1 periods clustered together, whereas those from the BR2 period began to show a dispersion trend. Over time, samples from the BR3, BR4 and BR5 periods showed more similar gene expression patterns. However, samples from the LE and BR6 periods showed a clear trend of separation from the other periods (Fig. 2A).

Analyses of differentially expressed genes between LB and FB, FB and BR2, BR2 and BR5 and BR5 and LE revealed 245 differentially expressed genes between LB and FB and 2,039 differentially expressed genes between FB and BR2. A total of 3,769 differentially expressed genes were found between BR2 and BR5 (Fig. 2B). Subsequently, these 37,842 differentially expressed genes were analyzed on the basis of clustering expression trends over time using STEM software. The results showed that these differentially expressed genes exhibited 50 different expression patterns within 7 periods. Among them, 20,709 differentially expressed genes showed 8 significantly clustered expression patterns (*p* < 0.05). The most critical of these patterns included expression pattern I, which showed downregulation, and expression pattern II, which showed downregulation followed by upregulation. In addition, differentially expressed genes in expression pattern 22 were upregulated from BR1 to BR2, downregulated from BR2 to BR3, and then upregulated from BR3 to BR4 and from BR5 to BR6, while expression pattern 8 showed a trend of downregulation followed by upregulation (Fig. 2C). *BgIAA8* was downregulated and then upregulated, *BgEIL1* was upregulated, and *BgIAA9* showed a decreasing trend. The *BgSCR* gene, a leaf development-related gene, was also expressed in bracts (Fig. S2).

GO enrichment analysis revealed that these differentially expressed genes were involved in important pathways, such as reproduction, reproductive processes and growth (Fig. 2D). The SBP and MADS-box families were the main families involved. Among them, members of the MADS-box family include *BgAP1*, *BgFULL*, and *BgCMB1*, while members of the SBP family include *SPL16*, *SPL8*, and *SPL14*. (Fig. 2E, F, G). These results further revealed the important role of gene expression regulation in plant bract development. KEGG enrichment analysis revealed that the metabolic pathways related to bract development were mainly related to porphyrin and chlorophyll metabolism, plant hormone signal transduction and other metabolic pathways (Fig. 2H, Fig. S3).

### Chlorophyll metabolic pathways during bract development

**Fig. 3:**
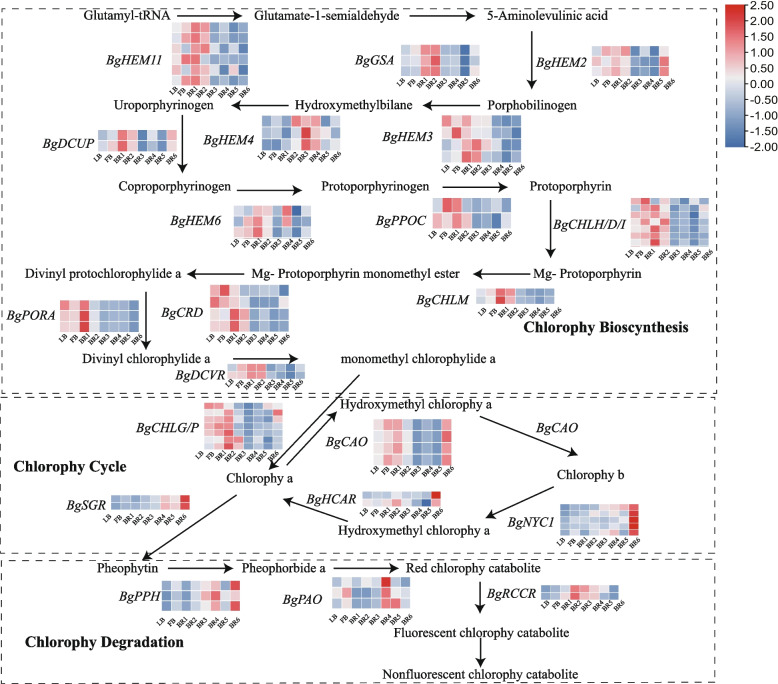
Chlorophyll metabolism pathway. *BgHEM11* (Glutamyl-tRNA reductase 1); *BgGSA* (Glutamate-1-semialdehyde 2,1-aminomutase); *BgHEM2* (Delta-aminolevulinic acid dehydratase); *BgDCUP* (Uroporphyrinogen decarboxylase); *BgHEM4* (Uroporphyrinogen-III synthase); *BgHEM3* (Porphobilinogen deaminase); *BgHEM6* (Oxygen-dependent coproporphyrinogen-III oxidase); *Bgppoc* (Protoporphyrinogen oxidase 1); *BgCHLH* (Magnesium-chelatase subunit *CHLH*); *BgCHLD* (Magnesium-chelatase subunit *CHLD*); *BgCHLI* (Magnesium-chelatase subunitI); *BgCHLM* (Magnesium protoporphyrin IX methyltransferase); *BgCRD* (Dicarboxylate diiron protein); *BgPORA* (Protochlorophyllide oxidoreductase A); *BgDCVR* (Divinyl chlorophyllide a 8-vinyl-reductase); *BgCHLG* (Chlorophyll synthase); *BgCHLP* (Geranylgeranyl reductase); *BgCAO* (Chlorophyllide a oxygenase); *BgSGR* (STAY-GREEN protein); *BgHCAR* (Coenzyme F420 hydrogenase family / dehydrogenase); *BgNYC1* (NAD(P)-binding Rossmann-fold superfamily protein); *BgPPH* (Pheophytinase); *BgPAO* (Pheophorbide a oxygenase, chloroplastic); *BgRCCR* (Accelerated cell death)

The color of *B. glabra* 'Mrs. Eva White' bracts changes from green to white during their development, a transition that is regulated by chlorophyll metabolism. Chlorophyll metabolism consists of three main processes: synthesis, recycling and degradation. In the synthetic pathway, hydroxymethyl chlorophyll is gradually reduced to chlorophyllide by the catalysis of *NADPH*-*POR*. *BgPORA* was highly expressed in the early stage of bract development, and its expression gradually decreased to very low levels as development progressed, suggesting that the ternary complex formed by hydroxymethyl chlorophyll a, *NADPH*, and *BgPOR* is the key to the green color of bracts in the early stage of bract development (Fig. 3). The genes found to interact with *BgPORA* by differential protein network analysis were *BgPPOC*, *BgPAO*, and *BgCHLH* (Fig. S4). The expression of the *BgSGR*, *BgPPH*, *BgPAO* and *BgRCCR* genes decreased at the early stage of bract development but then gradually increased, especially at the BR3 and BR4 stages. Therefore, it can be hypothesized that this period is the critical period for change in bracts color from green to white.

### Metabolism of auxin and abscisic acid during bract development

Endogenous plant hormones play an important role in leaf development and regulate the development of *B. glabra* bracts. Further in-depth analysis of the plant hormone signaling pathways led to the identification of many differentially expressed genes involved in hormone signaling response and transduction at key sites of bract formation. These genes are associated with a variety of hormones, such as auxin, abscisic acid, cytokinin, gibberellins, ethylene, and jasmonic acid.

**Fig. 4:**
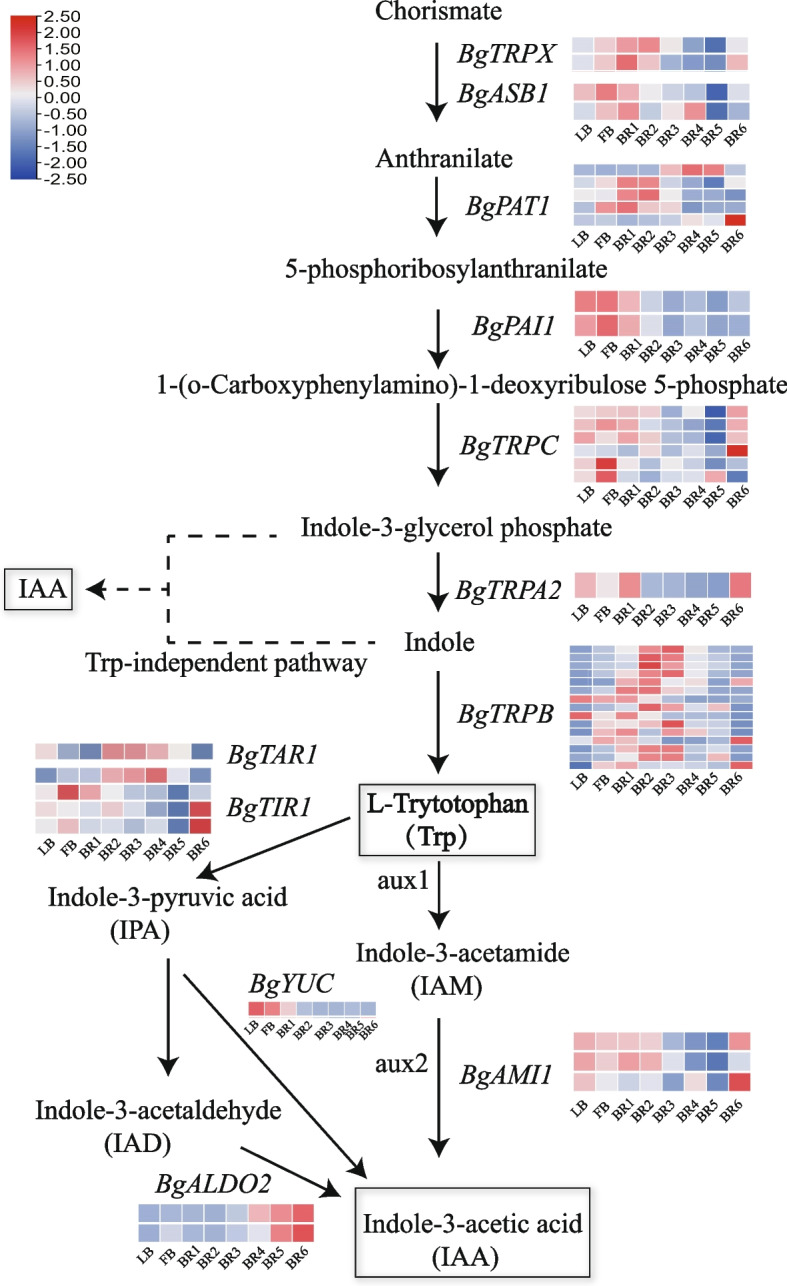
Expression analysis of plant growth hormone synthesis genes in *B. glabra* bract development stages. *BgTRPX* (Anthranilate synthase alpha subunit 2); *BgASB1* (Anthranilate synthase beta subunit 1); *BgPAT1* (Scarecrow-like protein 21 isoform X2); *BgPAI1* (Phosphoribosylanthranilate isomerase 1); *BgTRPC* (Indole-3-glycerol phosphate synthase); *BgTRPA2* (Tryptophan synthase alpha chain isoform X1); *BgTRPB* (Hypothetical protein); *BgTAR1* (Tryptophan aminotransferase related 1); *BgTIR1* (Transport inhibitor response 1-like); *BgYUC* (Probable indole-3-pyruvate monooxygenase); *BgAMI1* (Amidase 1); *BgALDO2* (Fructose-bisphosphate aldolase)

Indole-3-acetic acid (IAA) synthesis can be divided into the tryptophan-dependent pathway and the tryptophan-independent pathway. In the growth hormone signal transduction pathway, we further identified 44 homologous genes that encode 12 enzymes in the growth hormone synthesis pathway. The tryptophan-independent pathway includes 1 *BgASB1* gene, 1 *BgTRPX* gene, five *BgPAT1* genes, two *BgPAI1* genes, and six *BgTRPC* genes. The *BgTRPX*, *BgASB1*, *BgPAT1*, *BgPAI1*, *BgPRPC*, and *BgTRA2* genes in this pathway are highly expressed in the early stages of bract development and decrease in the later stages. The tryptophan-dependent pathway includes 15 *BgTRPB* genes, 4 *BgTIR1* genes, 3 *BgAMI1* genes, and 1 each of the *BgALDO2*, *BgTAR1*, *BgYUC*, and *BgTRPA2* genes. The expression of the *BgYUC* and *BgAMI1* genes of this pathway increased in the early stage. The expression of the *BgYUC* gene started to decrease gradually in the BR2 period, while that of the *BgAMI1* gene started to decrease gradually in the BR3 period, and the expression of the *BgALDO2* gene increased with bract development. These results suggest that these genes may play important regulatory roles in bract development (Fig. 4).

**Fig. 5:**
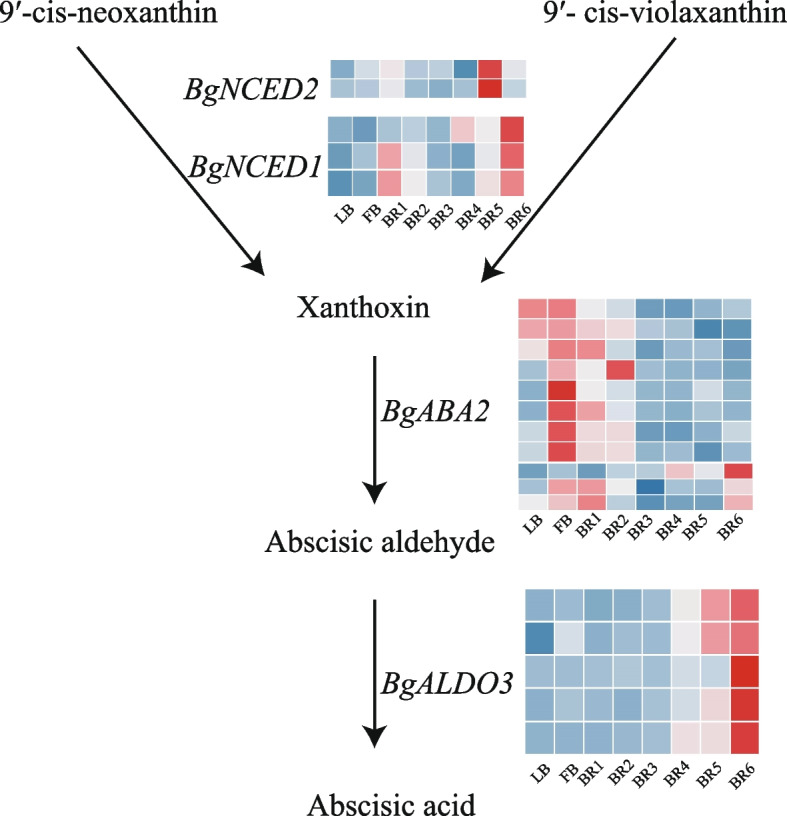
Expression analysis of abscisic acid synthesis genes in *B. glabra* bracts at different developmental stages. *BgNCED2* (Nine-cis-epoxycarotenoid dioxygenase 2); *BgNCED1* (9-cis-epoxycarotenoid dioxygenase); *BgABA2* (NAD(P)-binding Rossmann-fold superfamily protein); *BgALDO3* (Aldolase C)

In the abscisic acid signaling pathway, we further identified 23 homologous genes encoding four enzymes involved in the growth hormone synthesis pathway. These genes included 2 *BgECED2* genes, 3 *BgECED1* genes, 5 *BgALDO3* genes, and 12 *BgABA2* genes. *BgNCED2* was upregulated during BR5, and the *BgNCED1* and *BgALDO3* genes were upregulated during BR6. The *BgABA2* gene was upregulated during early development. Therefore, it is inferred that these genes play important regulatory roles in bract senescence (Fig. 5).

### Weighted gene correlation network analysis (WGCNA)

**Fig. 6:**
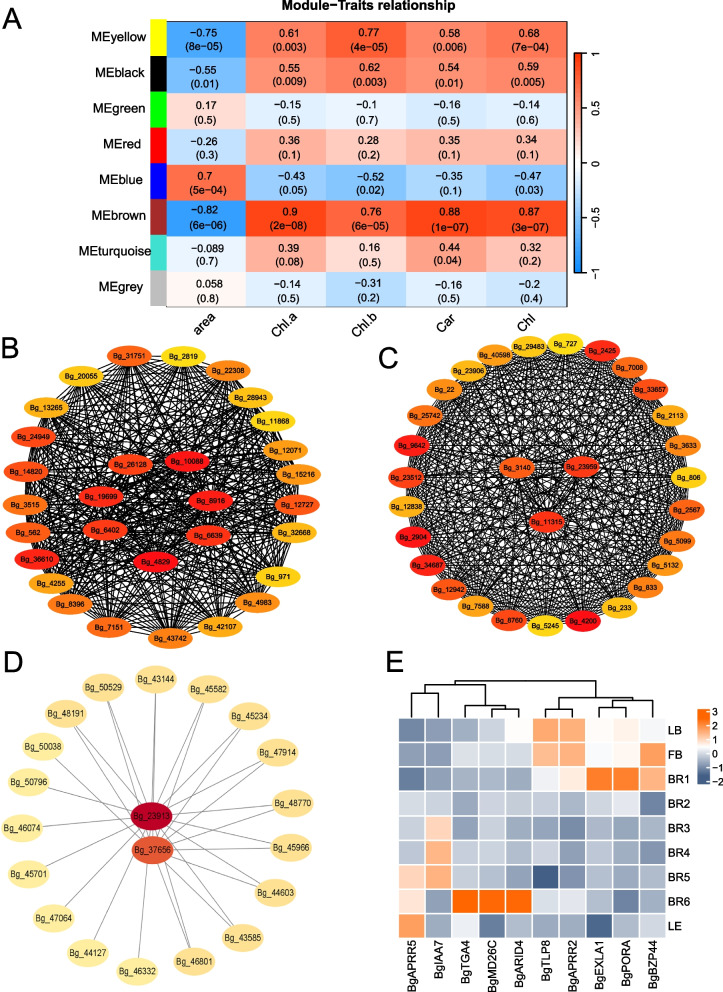
Weighted correlation network analysis of transcripts. **A** WGCNA with module-physiological growth indicator associations. Each row corresponds to a module. The names of the modules are shown on the left. Each column corresponds to a specific physiological growth indicator. The color of each cell at the row-column intersection indicates the correlation coefficient between the module and the sample. High correlations between specific modules and samples are indicated in red. **B** Interaction diagram of the gene network of the coexpressed genes of the ME blue module transcription factor HUB. **C** ME brown module transcription factor HUB gene network interaction plot. **D** Interoperation diagram of the ME brown module structural gene HUB gene network. **E** Heatmap of related HUB gene expression

A systems biology approach using weighted gene correlation network analysis (WGCNA) was applied, using 12,370 genes (FPKM ≥ 10) and 1,506 transcription factors, as a way to reveal the function of the network. In this network, the blue and brown modules showed a significant negative correlation with bract area and chlorophyll content, while the variability was particularly significant for the red and brown modules (Fig. 6A, Fig. S5).

Several key HUB genes were identified, including *Bg_3140*, *Bg_11315*, and *Bg_23959* in MEblue (Fig. 6B, Fig. S6); the transcription factor HUB genes *Bg_26128* and *Bg_10088* in MEbrown (Fig. 6C, Fig. S7); and structural genes with HUBs, mainly *Bg_23913* and *Bg_37656* (Fig. 6D). Notably, the HUB genes *BgPORA* and *BgEXLA1* showed significant differences in expression during BR1. Among them, the HUB gene *EXLA1* encodes an expansion protein that plays an important role in plant cell wall relaxation and regulates the expansion of *B. glabra* bract cells. The HUB gene *POR*A, on the other hand, is a key gene in the process of chlorophyll metabolism and participates in the biosynthesis of light-sensitive pigments by regulating the expression of *POR*A, which in turn affects chlorophyll biosynthesis, which is consistent with chlorophyll degradation during bract development in *Trigonella foetidum* (Fig. 6D).

In addition, we also found that the brown module HUB genes included *BgBZP44, BgAPRR5* and *BgIAA7*, which are related to the regulation of phy B photoreceptors and the regulation of plant growth hormones. During the development of *B. glabra* bracts, *BgAPRR5* expression gradually increased, especially during the BR6 period (Fig. 6E). *BgTLP8*, *BgAPRR2*, *BgBZP44*, *BgEXLA1*, and *BgPORA*, which play important roles in the regulation of *B. glabra* bract development and senescence and other processes, were highly expressed in the blue module during the early stages of bract development.

### qRT‒PCR of bract development-related candidate genes

**Fig. 7:**
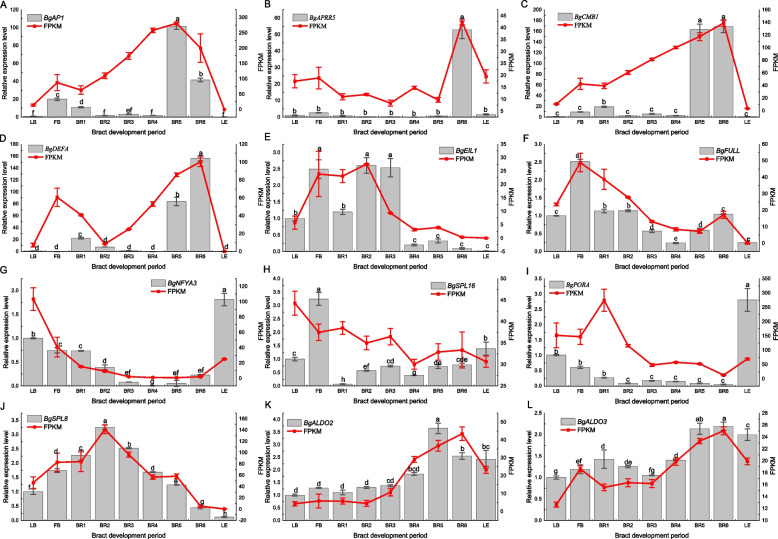
Candidate gene qRT‒PCR-identified FPKM values were derived from *B. glabra* transcriptome data. The actin gene was used as an internal control. LB as a control was assigned an arbitrary value of 1.0. The data represent three biological replicates and their mean values, and the error line represents the standard deviation of the three biological replicates

Based on the above analysis, we performed qRT‒PCR on three structural genes and nine transcription factors of the screened chlorophyll metabolic pathway. The qRT‒PCR results were also compared with the RNA-seq (FPKM) results to verify the consistency of the gene expression pattern with the sequencing results. The results were similar to the expression trends obtained by RNA-Seq (Fig. 7). By correlation analysis, *BgAP1* was found to be positively correlated with *BgCMB1*, *BgDEFA*, *BgALDO2*, *BgALDO3*, and *BgSPL8*, and *BgAP1* was inversely correlated with *BgNFYA3*, *BgPORA*, *BgAPRR5, BgSPL16*, and *BgFULL* (Fig. S8).

## Discussion

### Involvement of MADS-box proteins in* B. glabra* bract development

Flower development has been widely studied, but there are relatively few studies on bract development in ornamental plants, and the molecular mechanism underlying bract development is still unclear. MADS-box proteins are a large family of transcription factors that play key roles in eukaryotic development, regulating the intensity of target gene expression spatiotemporally by binding to cis-acting elements that interact with the target gene [15]. In this study, we found that the genes *BgAP1*, *BgCMB1*, *BgDEFA*, and *BgFULL*, which were differentially expressed during the development of *B. glabra* bracts, were all in the MADS-box family and that the expression of *BgAP1* was strongly inversely related to that of *BgFULL*. It has been suggested that *FRUITFULL* (*FULL)* is negatively regulated by *AP1* during the early stages of *Arabidopsis thaliana* development. *FULL* is hardly expressed in early trophic organs until after the onset of inflorescence development [16]. *AP1* represses *FULL* expression during sepal and petal development*.* AP3, on the other hand, inhibits the expression of *FULL* during stamen development [17]. Although it is unclear whether *FULL* is negatively regulated by *AP1* during bract development, it is hypothesized that mutual regulation of *BgAP1* and *BgFULL* may occur, based on their expression patterns in different developmental periods. Specifically, *BgAP1* was expressed mainly in the later stages of bract development, whereas *BgFULL* was expressed in the early stages of bract development. This finding is more consistent with the results of previous studies. While most of the MADS-box genes are expressed only in flowers, *FULL* is expressed at different specific stages of bract development. Investigations in a variety of plant species suggest that the requirement for B-class gene function during heterotopic petaloidy varies dramatically, and these genes may function early, late, or not at all during the development of petaloid organs [15, 18]. In *Aristolochia* (*Aristolochiaceae*, basal angiosperms), the expression of an *AP3-like* gene was detected only during the late stages of petaloid perianth development, and the expression of B-class paralogs was detected in the late development of *Aquilegia* petaloid sepals, suggesting that B-class homologs may only be required relatively late in the development of petaloid organs in these species [19].

*CMB1* is a gene of the MADS-box family that is involved in the regulation of pistil and sepal development in some plants. It has been suggested that *PpCMB1* regulates pistil and sepal development in *Prunus persica* [20]*. CMB1* is involved in fruit ripening and sepal development [21]. In *Solanum lycopersicum, SlCMB1* is a SEP-like MADS-box gene that participates in inflorescence and sepal development via interactions with other genes [22]. In this study, the expression of *BgCMB1* tended to increase with bract development and was positively correlated with *BgAP1* expression. *BgAP1, BgFULL, BgCMB1,* and *BgDEFA* were barely expressed in leaves but were highly expressed during bract development. These results suggest a potential role for *BgCMB1* in bract development, possibly because *BgCMB1* interacts with *BgAP1* and *BgFULL* to regulate bract development.

### The role of the SBP family in *B. glabra* bract development

SBPs are plant-specific class of transcription factors that are closely related to plant inflorescence development and yield [23]. SBPs have a zinc finger structure that recognizes and binds to the promoter of the MADS-box gene SQUAMOSA (SQUA), which is involved in plant growth and development as well as a variety of physiological and biochemical processes. In addition, SBPs, a class of transcription factors, can regulate flower and fruit development [24]. In *A. thaliana*, the SBP homologs *SPL3*, *SPL4* and *SPL5* regulate *A. thaliana* flower development, whereas the *SPL8* and *SPL14* genes regulate pollen development [25], which in turn affects yield.

The MADS-box transcription factor *FULL*, together with a SBP transcription factor, plays a role in reproductive transformation and meristem identity transformation [26]. *AtSPL3* in *A. thaliana* was identified as a direct upstream activator of *FULL,* and the activation of *FULL* by *SPL3* was very strong [27]. In *A. thaliana,* Yamasaki also demonstrated that *AtAP1* is downstream of the *AtSPL3* gene [28]. Further studies showed that after silencing and overexpressing the *PpSPL16* gene in *P. persica*, the expression of the *PpFULL* and *PpAP2a* genes was downregulated and upregulated, respectively, which further regulated fruit ripening in *P. persica* [29]. Whether *SPL16* interacts with *FULL* during bract development is unknown, but the analyses in this study revealed that both *BgSPL16* and *BgAP1,* as well as *BgSPL16*, play a crucial role in *B. glabra* bract development. In this study, the *BgSPL* genes exhibited different temporal and spatial expression patterns. The high expression levels of *BgSPL8*, *BgSPL13* and *BgSPL16* at the floral differentiation stage further confirmed the role of SPLs in regulating the asexual-to-reproductive transition through different regulatory roles during flower bud differentiation. *SPL3* does not play a major role in asexual changes or anthesis, but it promotes a shift in floral phloem identity by activating floral phloem-specific genes [30]. In addition, *SPL7* and *SPL8* induced phase transition and flowering in Gramineae by directly upregulating *SEPALLATA3* (*SEP3*) and *MADS32* [31]**.** *SPL8* was also found to be involved in GA signaling and positively regulated trichome formation on sepals and stamen filament elongation [32]. In this study, we found that *BgSPL8* was expressed in bracts but not in leaves during bract development, and *BgSPL16* was highly expressed during the FB period, with lower expression observed during other periods. Therefore, in the present study, we hypothesized that *BgSPL8* and *SPL16* are associated with bract development, which provides important insight for further studies on plant growth and development.

### Chlorophyll metabolism during bract development in *B. glabra*

In this study, *B. glabra* 'Mrs. Eva White' bract pigmentation was found to involve chlorophyll, carotenoids, and flavonoids, and at the beginning of the developmental stage, *B. glabra* bracts had a high chlorophyll content; however, as the bracts developed, the color gradually changed to white. Recently, phytochrome-mediated light signaling was reported to induce *PORA* gene expression in monocotyledons such as *Hordeum vulgare* and, similarly, in dicotyledons such as *A. thaliana * [33, 34]. Moreover, it has been suggested that *PORA* is abundant in yellowing plants, is active mainly during seedling deyellowing, and acts synergistically with the light-trapping complex [35]. A mutant of *A. thaliana* with yellow leaves that did not turn green when grown in white light was identified, confirming that the presence of *PORA* confers a certain degree of photoprotection to the plant. The reaction of *PORA* with pchlide is the only step in which a light-requiring enzyme mediates the biosynthesis of chlorophyll in higher plants [36], and its expression is regulated by photosensitive pigments, thus affecting chlorophyll biosynthesis. This study further revealed that *BgPORA* may be a key gene for chlorophyll formation during bract development.

In addition, the STAY-GREEN protein (SGR) plays key regulatory roles in chlorophyll degradation and functions independently of *PAO* enzymes. Moreover, alterations in the transcription levels of genes encoding chlorophyll-degrading enzymes, including *PPH*, *PAO* and *RCCR*, which have been identified in model crop species such as *Oryza sativa* and *A. thaliana*, affect the process of chlorophyll degradation and, consequently, the yellowing or senescence phenotypes of the leaves. It has been suggested that knockdown or inhibition of *SGR* expression delays chlorophyll degradation, leading to a stagnant green phenotype in plants during natural development or dark-induced senescence [37]. In this study, *B. glabra* bracts were found to be upregulated during development with *BgSGR* in the later stages of *B. glabra* bract development, and *BgSGR* may be a key gene regulating the whitening of green bracts.

### Effects of auxin and abscisic acid during bract development

Auxin is a phytohormone that plays an important role in plant growth and development. It has an unsaturated aromatic ring and an acetic acid side chain, in which indole-3-acetaldoxime can be converted to indole-3-acetaldehyde, and it can be catalytically converted to IAA by the enzyme *ALDO2*. This pathway is currently relatively understudied, especially with regard to bract development. The indole-3-pyruvic acid (IPA) pathway is the main pathway for IAA biosynthesis in plants and is common in most plant species [38]. It has been shown that *YUCCA* (*YUC*) is involved in the IPA pathway in synergy with tryptophan aminotransferase (TAA) of *Arabidopsis*, wherein TAA is involved in catalyzing the conversion of tryptophan to IPA, and subsequently, *YUC* is involved in catalyzing the production of IAA from IPA [39, 40]. The *YUC* and TAA families mainly regulate embryonic development after the spherical embryo stage [41]. In this study, we found that the *BgYUC* gene is highly expressed mainly during the prophase of the growth hormone synthesis pathway and plays a regulatory role in the prophase of bract development. In this study, we hypothesized that *BgYUC* expression in this pathway is high in the early stages of bract development and low in the later stages, but the underlying molecular mechanism in bract development remains to be investigated.

The abscisic acid metabolic pathway mainly includes the terpenoid pathway and the carotenoid pathway [42]. The terpenoid pathway, also known as the C15 direct pathway, consists of the direct formation of 15-carbon ABA from farnesyl pyrophosphate (FPP) via cyclization and oxidation, whereas the carotenoid pathway, also known as the C40 indirect pathway, is the main pathway for major *BgABA* synthesis in higher plants. In this pathway, the conversion of zeaxanthin to violaxanthin is catalyzed by zeaxanthin epoxidase (ZEP), which is followed by 9-cis-epoxy carotenoid dioxygenase (NCED)-mediated catalysis of the production of xanthoxin from 9-cis-neoxanthin. The short-chain alcohol dehydrogenase *BgABA2* is converted to ABA-aldehyde, which is ultimately converted to ABA by *ALDO3* [43]. In this study, we found that *BgALDO3* was highly expressed during the BR6 period at the end of bract development, suggesting that bract senescence is associated with Bg*ALDO3*. It has been suggested that the ABA signaling pathway is associated with pseudoresponse regulator 5 (*APRR5*) and pseudoresponse regulator 7 (*APRR7*) and that *ABI5*, a key regulator in the ABA signaling pathway, specifically interacts with *APRR5* and *APRR7* [44]. In this study, *BgAPRR5* was found to be highly expressed during the BR6 period, and it was hypothesized that this gene is associated with the abscisic acid metabolic pathway, which provides important clues for understanding the molecular mechanisms underlying the effects of plant auxin and abscisic acid in bract development.

## Conclusion

In conclusion, our investigation into the development of *B. glabra* bracts revealed critical physiological and genetic mechanisms involved. We observed key changes in bract morphology, including increased area and chlorophyll content, accompanied by a reduction in hairiness (glabrescence). These morphological changes strongly correlate with the dynamics of hormone transduction pathways and chlorophyll metabolism. Importantly, genes within the MADS-box and SBP families, such as *BgAP1*, *BgFULL*, *BgSPL16*, *BgCMB1*, and *BgDEFA*, play pivotal roles in these developmental processes. Our study also highlights the significance of the *BgPORA* and *BgSGR* genes in the biosynthesis and breakdown of chlorophyll during bract development. The expression patterns of *BgPRPC* and *BgTRA2* suggest their involvement in early developmental stages through both tryptophan-dependent and tryptophan-independent growth hormone pathways. Furthermore, the upregulation of *BgALDO3* during the BR6 period underscores its potential importance in later developmental stages, particularly in the abscisic acid pathway. These findings not only enhance our understanding of bract development in *B. glabra* but also suggest avenues for future research to explore the regulatory mechanisms of floral architecture in ornamental plants.

## Materials and methods

### Plant material and sample collection

*Bougainvillea glabra* 'Mrs. Eva White' planted at the flower base of Hunan Agricultural University was selected as the plant material for this study in August 2021, and the bract developmental stages were divided into eight stages for bract sample collection (Fig. 1A). Three plants were set up as 1 biological replicate, and 3 biological replicates were obtained at each developmental stage. Twenty representative samples were collected from each biological replicate and mixed. These samples were immediately placed in liquid nitrogen, stored at -80 °C for RNA extraction and then sent to Fraser Bioinformatics for sequencing using a PacBio sequencer.

### Microscopic observation, paraffin sectioning, and scanning *electron* microscopy

Dissection of bracts at the flower bud stage was performed to observe their internal structure. Buds from both the LB and FB periods were selected for paraffin sectioning. Samples from the LB and FB periods were soaked in FAA fixative, and the subsequent experiments were performed by Wuhan Safeway Biotechnology Co. To understand flower bud development via resin sectioning, buds from different periods were collected, immediately soaked in FAA fixative (70% ethanol:glacial acetic acid:formaldehyde: 90:5:5) and dehydrated after embedding using a Technovit 7100 resin embedding kit. After embedding, the resin was sectioned with a Leica EM UC6 microtome, and 0.1% toluidine blue dye solution was used for staining for microscopy. To observe the phenotypic changes in bract development, samples were taken from the BR1, BR3, BR5, and LE periods for electron microscopy scanning, and the subsequent experiments were performed at Wuhan Sevier Biotechnology Co.

### Bract area and chlorophyll measurement

*B. glabra* 'Mrs. Eva White' bracts exhibited an increase in area and faded from green to white during growth. The rate of bract development was observed daily, and the area of *B. glabra* 'Mrs. Eva White' bracts was measured using a leaf area meter. The chlorophyll and carotenoid contents of leaf buds, leaf blades and bracts at different periods of development were determined by ultraviolet‒visible spectrophotometry. Several samples were harvested, washed and cut into strips of approximately 2 mm, weighed to 0.2 g, and extracted in 95% ethanol for 24 h, and three replicates were set up for each species in each treatment. After 24 h, the absorbance at 470 nm, 665 nm and 649 nm was measured by an ultraviolet‒visible spectrophotometer, and the contents of chlorophyll a, chlorophyll b and carotenoids were calculated.

### *Iso*-Seq analysis method

Total RNA was extracted from the tissue samples. The concentration and purity of the extracted RNA were tested by a Nanodrop 2000; the genomic contamination, purity and integrity of the RNA samples were assessed by agarose gel electrophoresis; and the RNA integrity number (RIN) values were determined by an Agilent 2100. High-quality RNA is the basis for successful sequencing. To ensure the accuracy of the sequencing data, the samples were tested to ensure that they met requirements before library construction. After library testing, full-length transcriptome sequencing was performed using a PacBio sequencer according to the target downstream data volume. The raw sequencing data were preprocessed using SMRTlink software, and full-length transcript sequences were obtained via Iso-Seq analysis.

### STEM and PCA

Gene expression levels were calculated as the number of exon model reads per kilobase per million mapped reads (FPKM). Based on the FPKM data of the differentially expressed genes at the seven bract developmental stages, the expression trends of the differentially expressed genes over time were analyzed by clustering using STEM software. PCA of the dataset was performed using the OmicShare Tools tool (https://www.omicshare.com/tools/home/report/reportpca.html).

### Identification of coexpression modules and visualization of hub genes

Based on the gene-level FPKM data of differentially expressed genes at the seven bract developmental stages, the R software package for WGCNA was used [45]. The default minimum number of genes per module was 30, and the threshold for module merge correlation was 0.9 (Fig. S9). These modules were obtained using the automated network construction function block with default settings. Eigenvalues were calculated for each module and used to test for associations with area and chlorophyll content. The genes were grouped into 8 specific modules. The network was visualized using Cytoscape_v.3.8.0 [46].

### Quantitative real-time PCR

The BgActin gene was used as the internal control. The primers used in the study are listed in Supplemental Table S1. Real-time fluorescence quantitative RT‒PCR was performed using a ChamQ Universal SYBR qPCR Master Mix kit. The relative gene expression levels were normalized using the 2^−ΔΔCt^ method.

### Data analysis

The experimental results are expressed as the mean ± standard error and were analyzed using Excel 2010 and SPSS 22.0. The significance of differences among different datasets was analyzed using Duncan's multiple range test at a significance level of *p* < 0.05. Heatmaps for this article were generated with OmicShare Tools (https://www.omicshare.com/tools/home/report/reportpca.html). Real-time fluorescence quantitative RT‒PCR plots were generated with Origin software.

### Supplementary Information

Additional file 1: Table S1. Primers used in this study

Additional file 2: Fig. S1. A Resin section of the* B. glabra* bud development process. (a) Undifferentiated period. (b) Floret primordial differentiation period. (c) Whorls around the primordial perianth. B Paraffin sections of leaf buds and flower buds. (a) Paraffin section from the LB period. (b) Paraffin section from the FB period. C. Electron microscopy image of the development of* B. glabra *bracts. (a) *B. glabra* bracts from the BR1 period. (b)* B. glabra *bracts from the BR3 period. (c) *B. glabra *bract from the BR5 period.

Additional file 3: Fig. S2. Differential gene expression heatmap

Additional file 4: Fig. S3. Significantly enriched KEGG metabolic pathways at different developmental stages are represented by bubble plots. A FB-VS-BR1. B BR2-VS-BR3. CBR1-VS-BR5

Additional file 5: Fig. S4. Differential protein network interaction map.

Additional file 6: Fig. S5. Gene module partition map.

Additional file 7: Fig. S6. Heatmap of gene expression patterns and bar plot of the eigengene expression levels in the blue module.

Additional file 8: Fig. S7. Heatmap of gene expression patterns and bar plot of the eigengene expression levels in the brown module.

Additional file 9: Fig. S8. Correlation analysis of gene FPKM values with RT‒qPCR

Additional file 10: Fig. S9. Gene scale independence and average connectivity of different powers under the assumption of scaleless networks.

## Data Availability

The sequencing data associated with transcription profiles in this study have been deposited in the China National Center for Bioinformation database with accession number GSA:CRA006581( https://bigd.big.ac.cn/GSA/browse/CRA006581).

## Abbreviations

LB: Leaf bud
FB: Floral bud
BR: Bract
LE: Leaf
WGCNA: Weighted correlation network analysis
IAA: Indole-3-acetic acid
IPA: Indole-3-pyruvic acid
SBP: Squamosa promoter binding protein
PCA: Principal component analysis
*AP1*: APETALA1
*FULL*: FRUITFULL
*HEM11*: Glutamyl-tRNA reductase 1
*GSA*: Glutamate-1-semialdehyde 2,1-aminomutase
HEM2: Delta-aminolevulinic acid dehydratase
*DCUP*: Uroporphyrinogen decarboxylase
*HEM4*: Uroporphyrinogen-III synthase
*HEM3*: Porphobilinogen deaminase
*HEM6*: Oxygen-dependent coproporphyrinogen-III oxidase
*POOC*: Protoporphyrinogen oxidase 1
*CHLH*: Magnesium-chelatase subunit
*CHLD*: Magnesium-chelatase subunit
*CHLI*: Magnesium-chelatase subunitI
*CHLM*: Magnesium protoporphyrin IX methyltransferase
*CRD*: Dicarboxylate diiron protein
*PORA*: Protochlorophyllide oxidoreductase A
*DCVR*: Divinyl chlorophyllide a 8-vinyl-reductase
*CHLG*: Chlorophyll synthase
*CHLP*: Geranylgeranyl reductase
*CAO*: Chlorophyllide a oxygenase
*SGR*: STAY-GREEN protein
*HCAR*: Coenzyme F420 hydrogenase family/dehydrogenase
*NYC1*: NAD(P)-binding Rossmann-fold superfamily protein
PPH: Pheophytinase
CHLH: Pheophorbide a oxygenase, chloroplastic
RCCR: Accelerated cell death
*TRPX*: Anthranilate synthase alpha subunit 2
*ASB1*: Anthranilate synthase beta subunit 1
*PAT1*: Scarecrow-like protein 21 isoform X2
PAI1: Phosphoribosylanthranilate isomerase 1
*TRPC*: Indole-3-glycerol phosphate synthase
*TRPA2*: Tryptophan synthase alpha chain isoform X1
TRPB: Hypothetical protein
*TAR1*: Tryptophan aminotransferase related 1
*TIR1*: Transport inhibitor response 1-like
*YUC*: Probable indole-3-pyruvate monooxygenase
*AMI1*: Amidase 1; *ALDO2*: fructose-bisphosphate aldolase
*NCED2*: Nine-cis-epoxycarotenoid dioxygenase 2
*NCED1*: 9-Cis-epoxycarotenoid dioxygenase
*ABA2*: NAD(P)-binding Rossmann-fold superfamily protein
*ALDO3*: Aldolase C
