# Supplementary material for: Transcriptomic profiling and gene network analysis revealed regulatory mechanisms of bract development in Bougainvillea glabra
Source: BMC Plant Biol. 2024 Jun 13;24:543. doi: 10.1186/s12870-024-05246-7 (PMC11177516; doi:10.1186/s12870-024-05246-7)
Supplement: Supplementary file 1 — Additional file 1: Table S1. Primers used in this study [file 12870_2024_5246_MOESM1_ESM.docx]

Table S1. Primers used in this article

| Primer name | Primer sequence（5’ to 3’） |
| --- | --- |
| Bgactin-F | TAGACCCTCCTATCCAAACA |
| Bgactin-R | TTTTCCAGCCTTCACTTATC |
| BgAP1-F | AGCAGCAACTTAACAATG |
| BgAP1-R | TCAATCTTCTCCTTATCCTT |
| BgAPRR5-F | TGCTGCTCAACTTCGTAA |
| BgAPRR5-R | TATTGTGCTTCCGCTTCA |
| BgCMB1-F | GAATATCTGAAGTTGAAGG |
| BgCMB1-R | TGTGTTGTGTCTTAATTG |
| BgDEFA-F | ACTAATAGACAGGTGACT |
| BgDEFA-R | CAACATCCATCATCTTCT |
| BgEIL1-F | CTCATTGTCCTCATAATCA |
| BgEIL1-R | TCCATCTGTAAGTTGTTG |
| BgFULL-F | GAGAATAAGATAAGTCGTCAAGT |
| BgFULL-R | TCGCACAGAACAGAGATT |
| BgNFYA3-F | ATCATCCAGTCAATCAAC |
| BgNFYA3-R | CATTCTTAGCACAACTCA |
| BgSPL16-F | CATAAGGTCTGTGAGGTT |
| BgSPL16-R | ATCATCTTCTTCAATAATAAATCC |
| BgPORA-F | CTCACAATGCAGGAGTTC |
| BgPORA-R | CTCTGAACAAGCCAGTAG |
| BgALDO2-F | TCCATTGTTGTAGAAGTA |
| BgALDO2-R | TATCTGCCTGTATAACTC |
| BgALDO3-F | CCAGGCTCCAGAATATAC |
| BgALDO3-R | AGTAATGATGCGAATGTTG |
| BgSPL8-F | AACAACAGCAACAACAAG |
| BgSPL8-R | GGTGACTTAGGAGGAATATC |


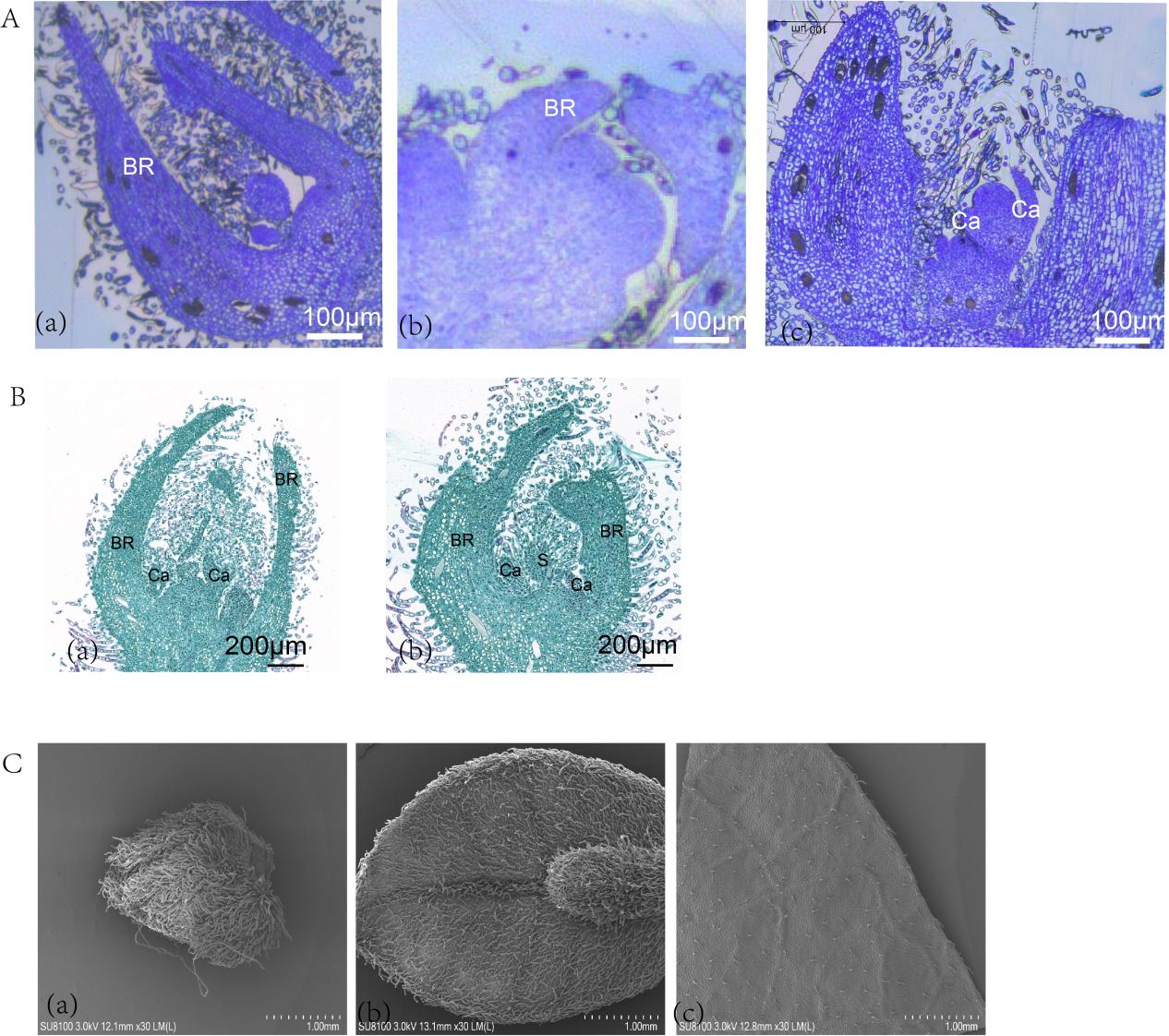


Fig*.S*1 A Resin section for *B. glabra* bud development process (a) undifferentiated period (b) floret primordial differentiation period .(c) whorls around the primordial perianth, Fig. B Paraffin sections of leaf buds and flower buds .(a) paraffin section at LB period. (b) paraffin section at FB period. Fig. C is an electron microscope scan of the development of *B. glabra* bracts at (a) B.glabra bracts at the BR1 period. (b) *B.glabra* bracts at the BR3 period. (c) *B .glabra* bract BR5 period.


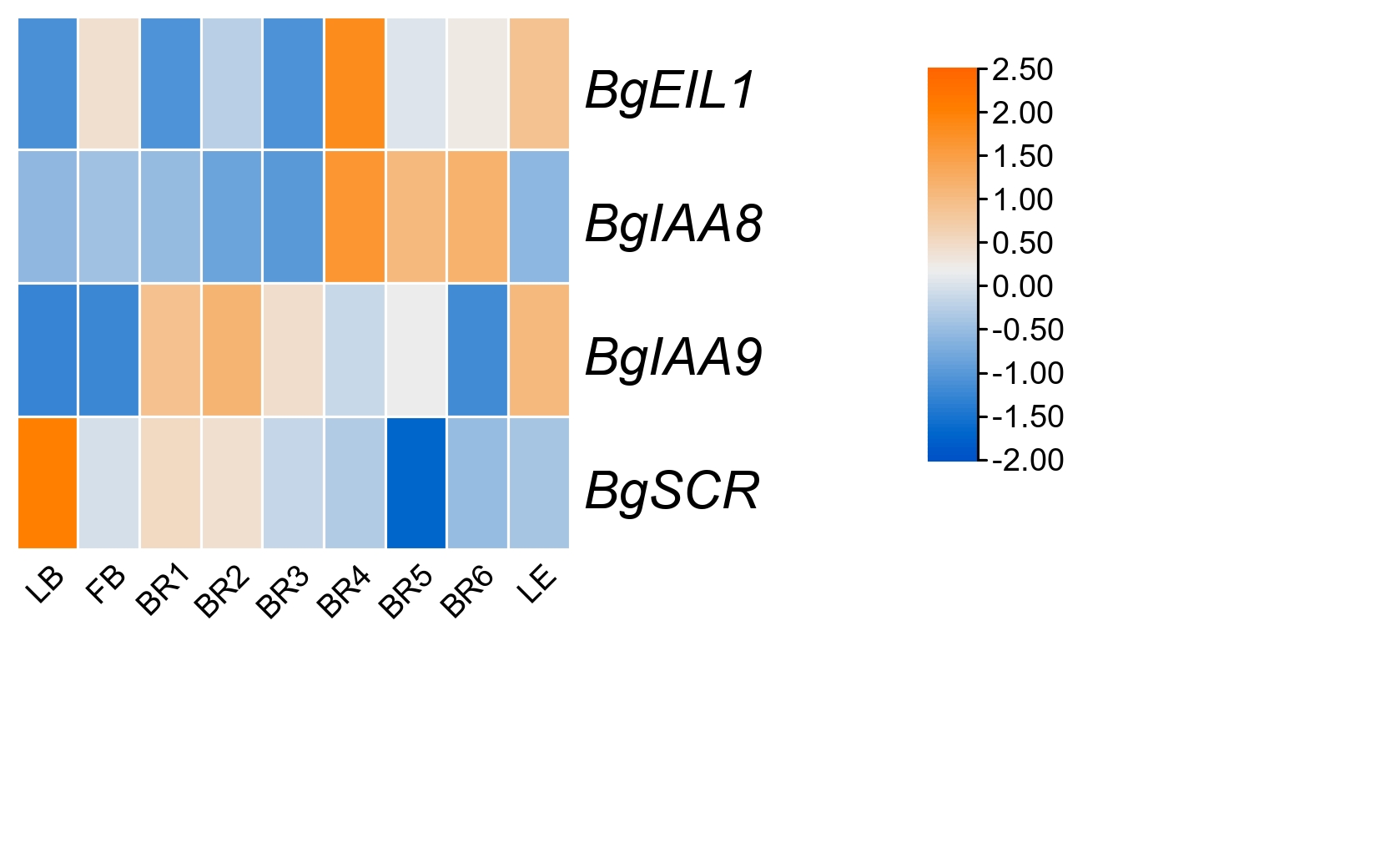


Fig.S2 Differential Gene Heat Map


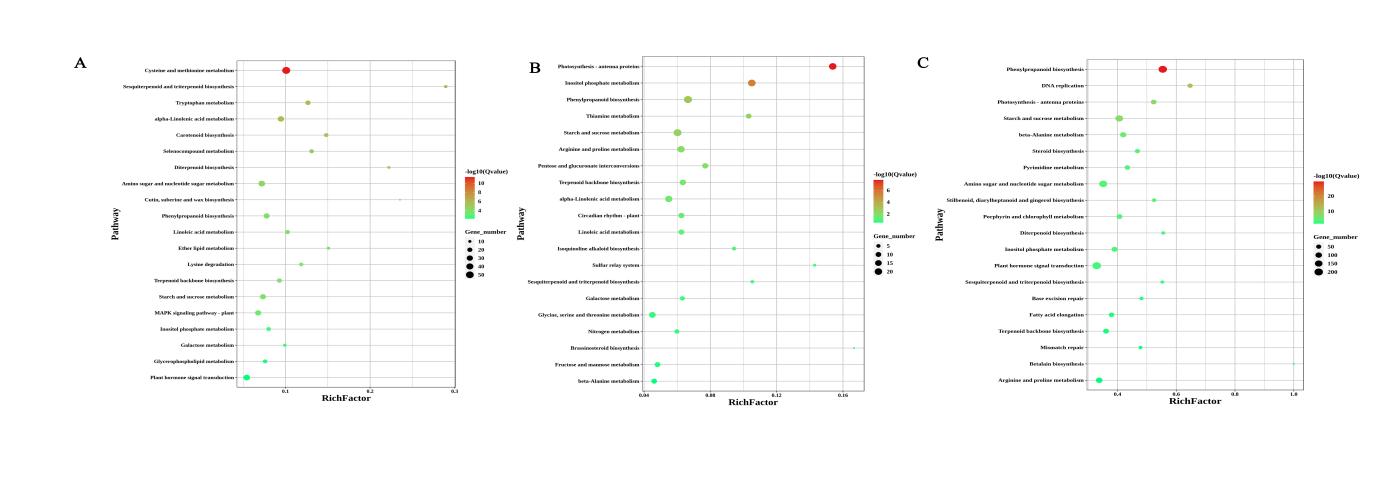


Fig.S3Significantly enriched KEGG metabolic pathways at developmental stages are represented by bubble plots.A Fig. FB-VS-BR1 period,B Fig. BR2-VS-BR3 period,C Fig. BR1-VS-BR5


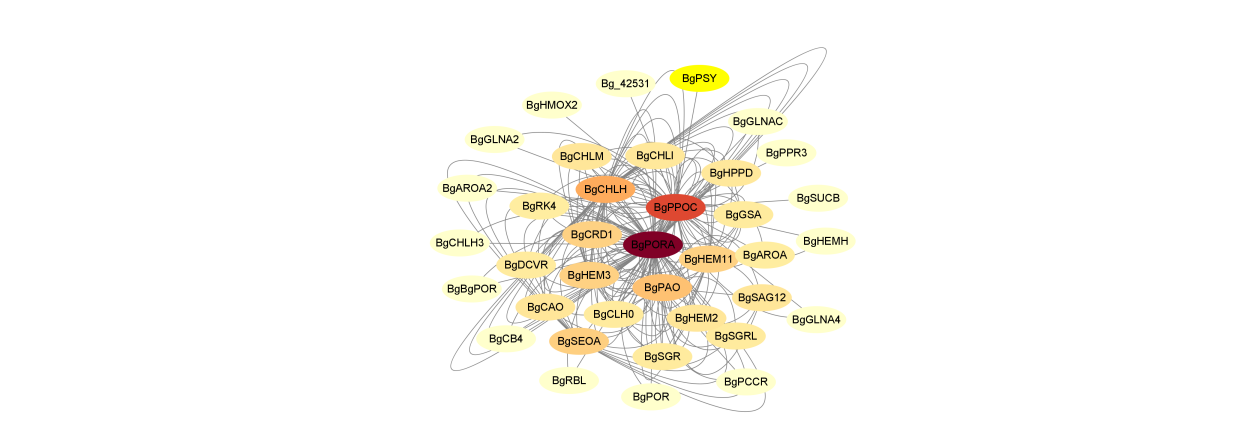


Fig.S4 Differential protein network interaction map


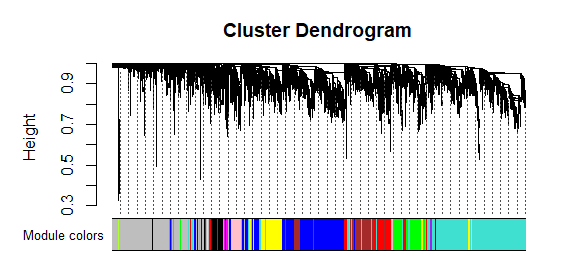


Fig.S5 Gene module partition map


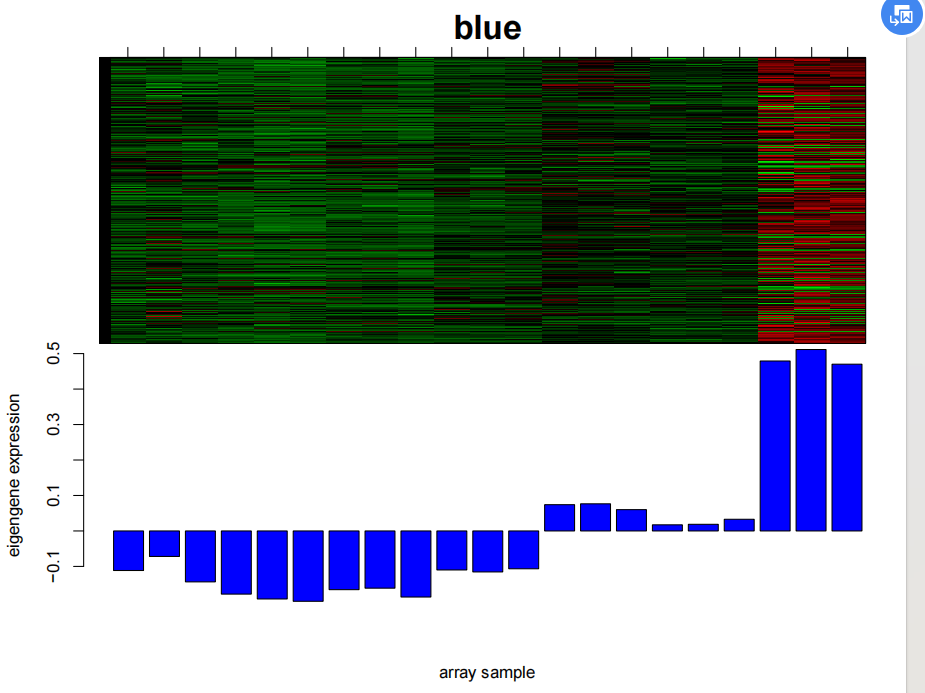


Fig.S6 Heatmap of gene expression pattern and bar plot of eigengen expression of genes in blue

module


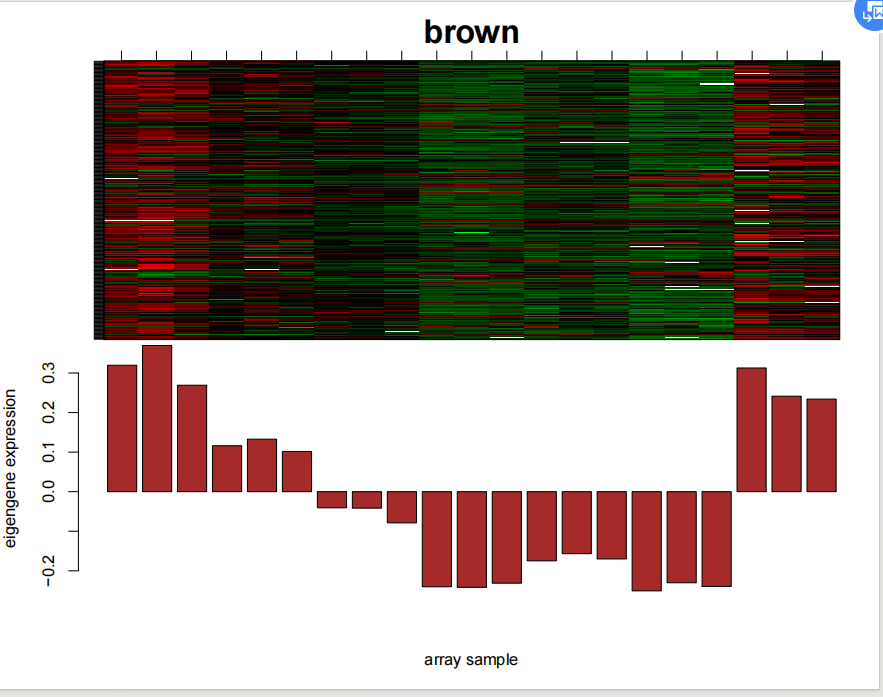


Fig.S7 Heatmap of gene expression pattern and bar plot of eigengen expression of genes in brown

Module


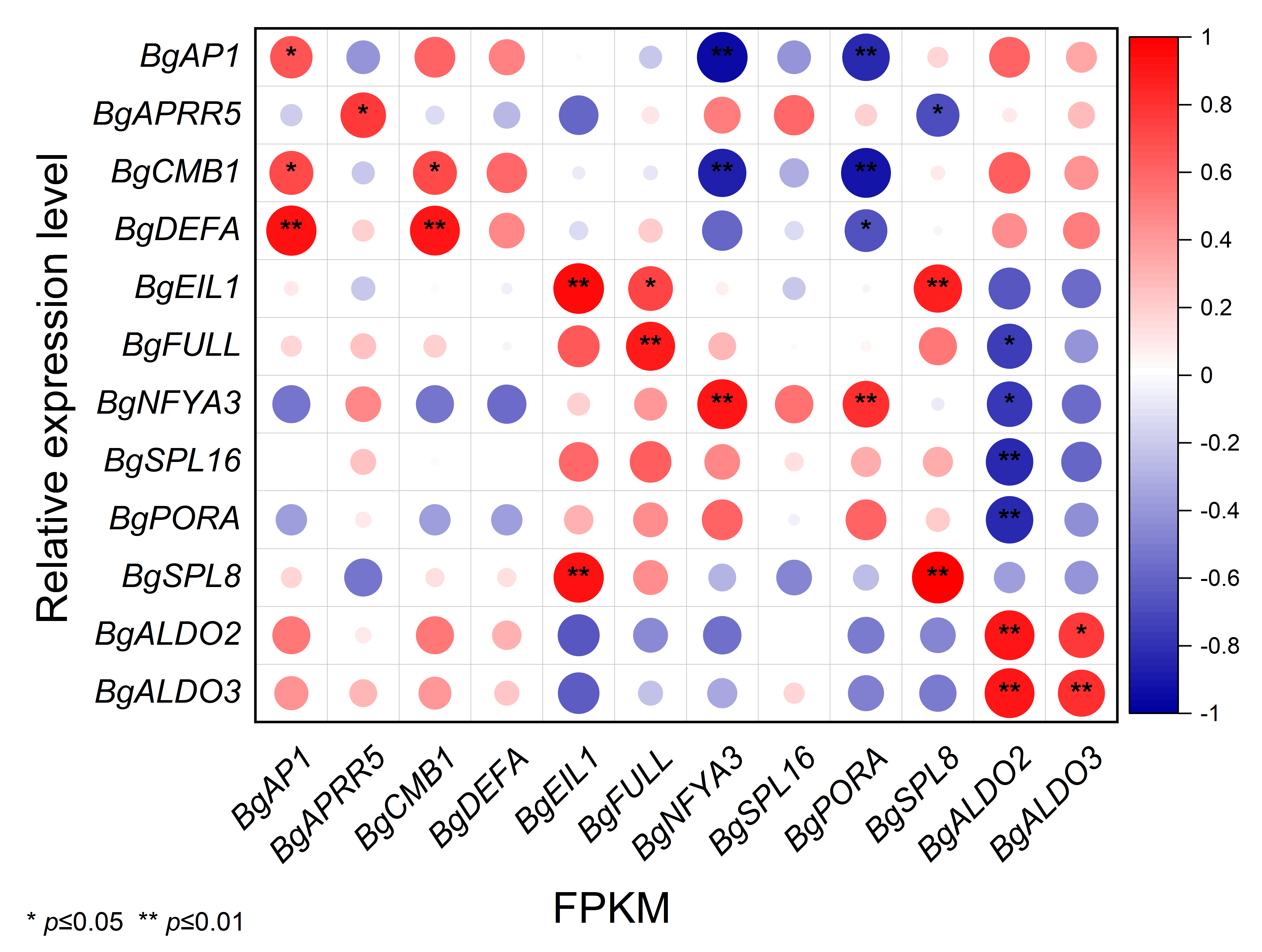


Fig.S8 Correlation analysis of gene FPKM values with RT-qPCR


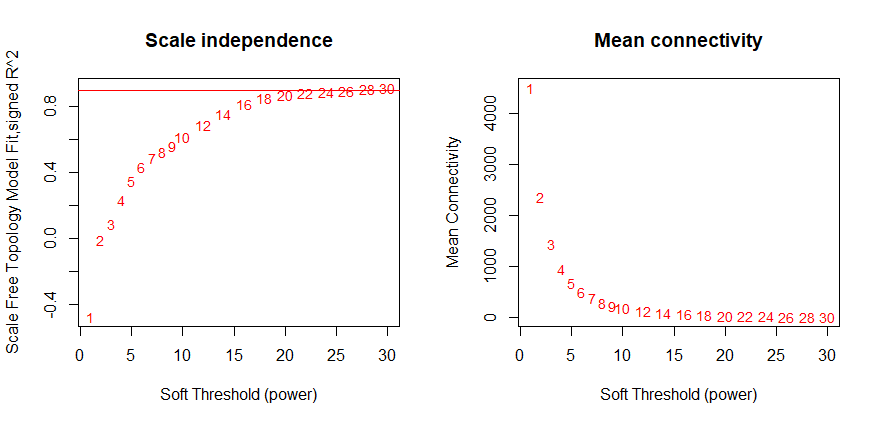


Fig.S9 Gene scale independence and average connectivity of different powers under the

assumption of scaleless networks
