# Supplementary figures and images for: Transcriptomic profiling and gene network analysis revealed regulatory mechanisms of bract development in Bougainvillea glabra
Source: BMC Plant Biol. 2024 Jun 13;24:543. doi: 10.1186/s12870-024-05246-7 (PMC11177516; doi:10.1186/s12870-024-05246-7)

A

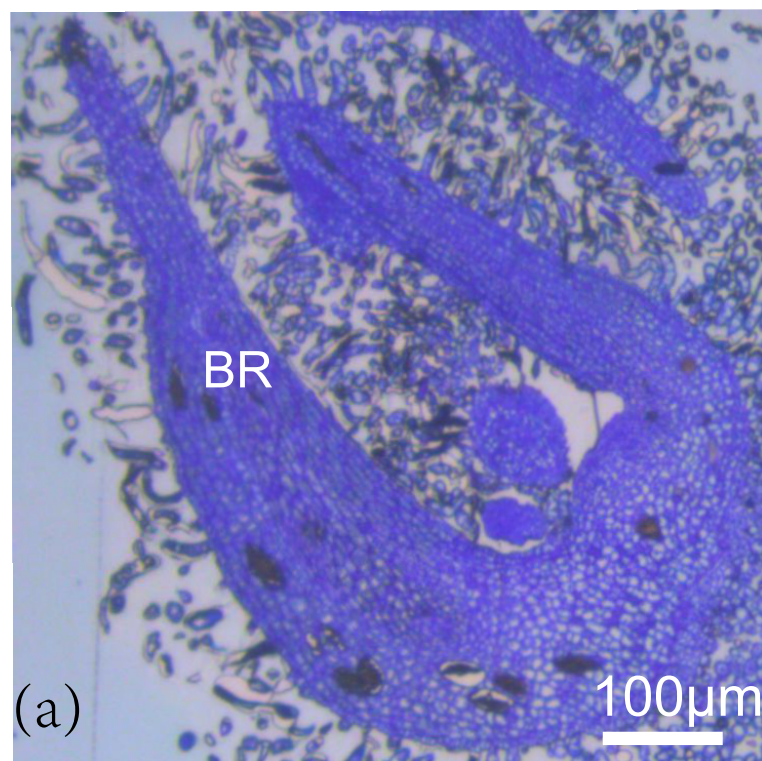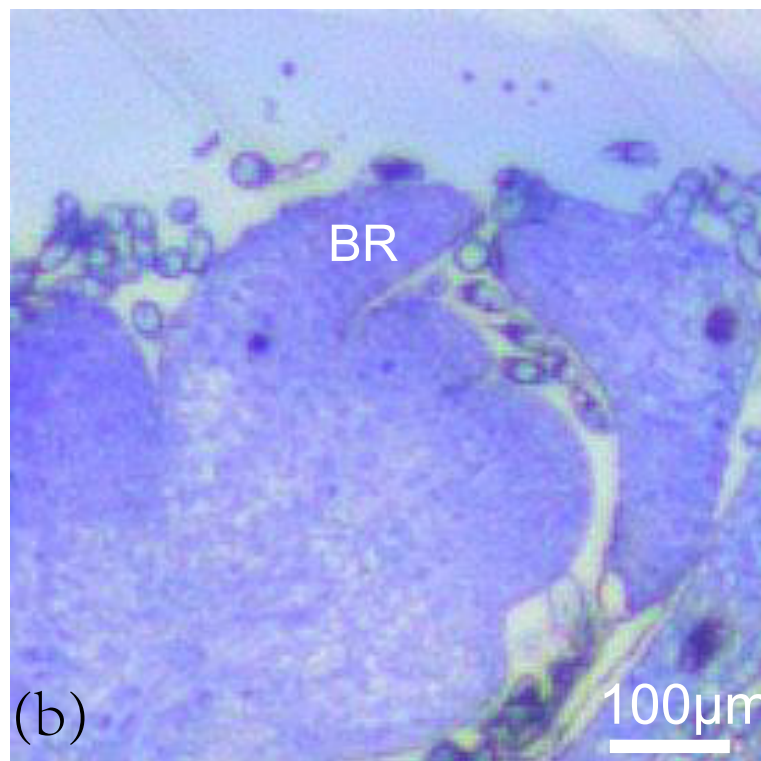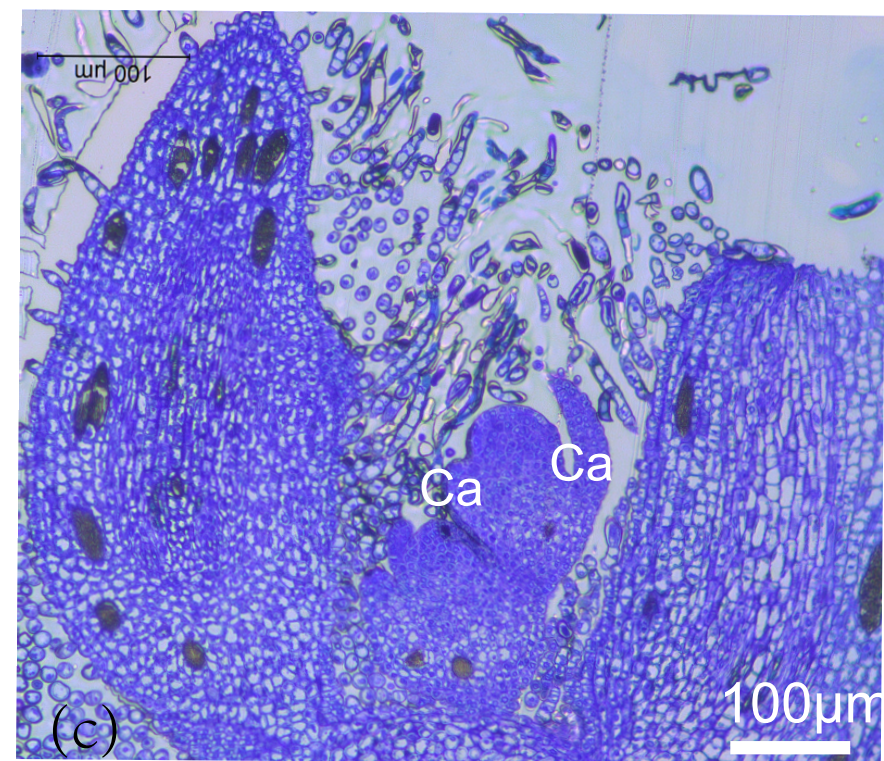

B

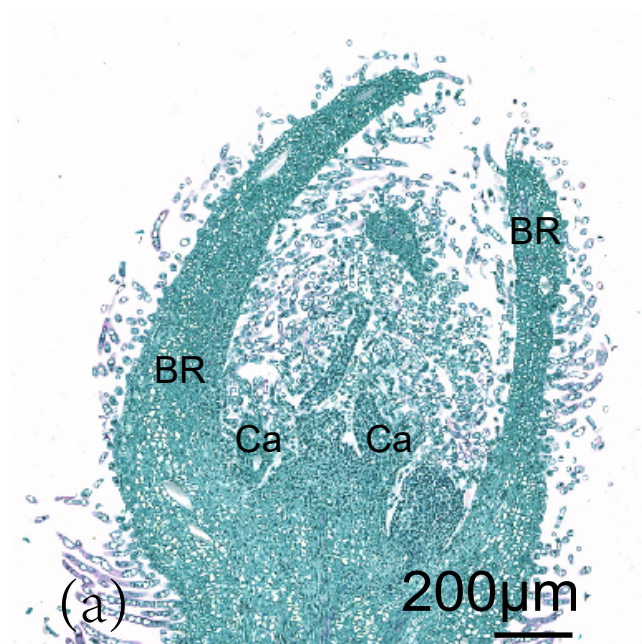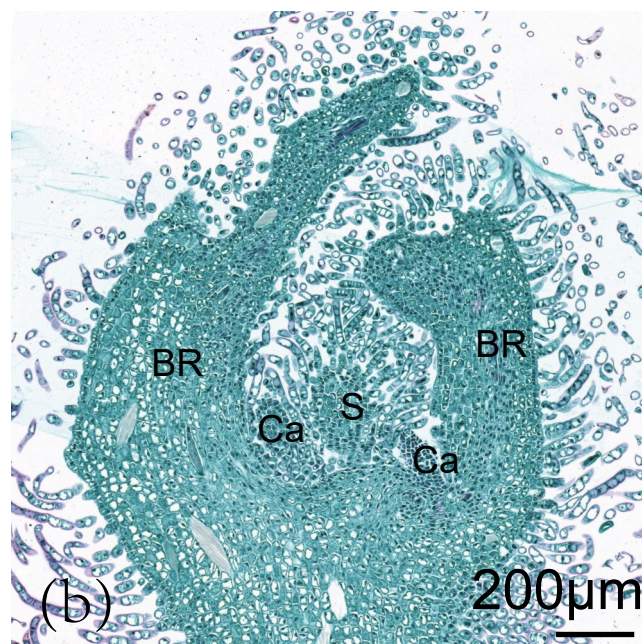

C

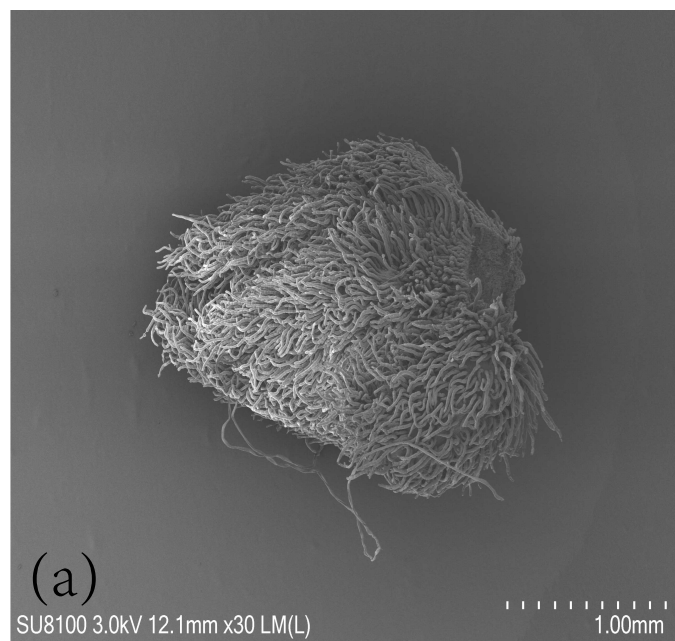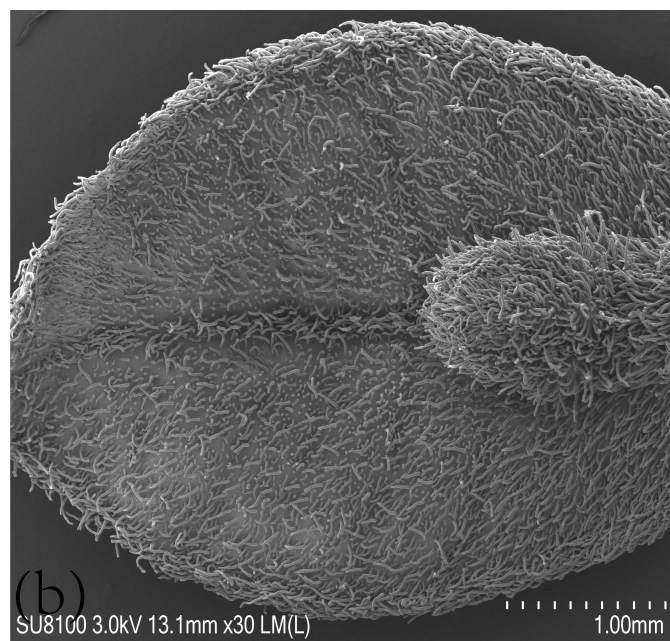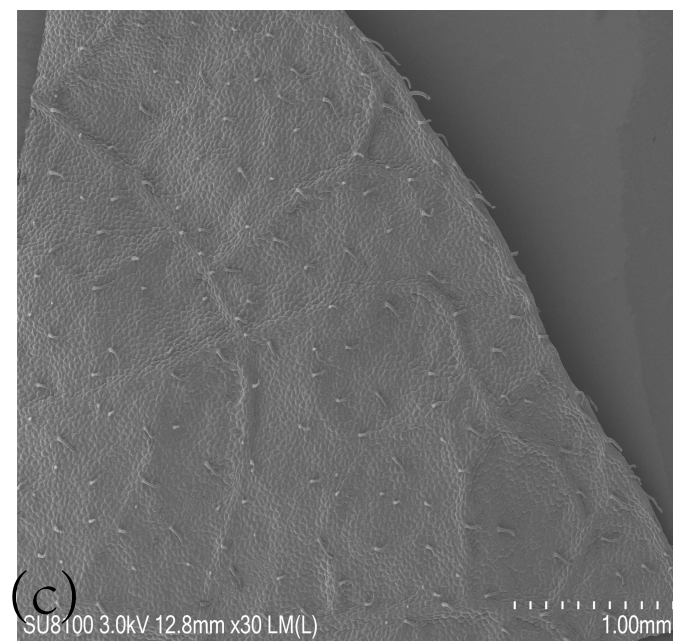

Supplement: Supplementary file 2 — Additional file 2: Fig. S1. A Resin section of the B. glabra bud development process. (a) Undifferentiated period. (b) Floret primordial differentiation period. (c) Whorls around the primordial perianth. B Paraffin sections of leaf buds and flower buds. (a) Paraffin section from the LB period. (b) Paraffin section from the FB period. C. Electron microscopy image of the development of B. glabra bracts. (a) B. glabra bracts from the BR1 period. (b) B. glabra bracts from the BR3 period. (c) B. glabra bract from the BR5 period. [file 12870_2024_5246_MOESM2_ESM.pdf]

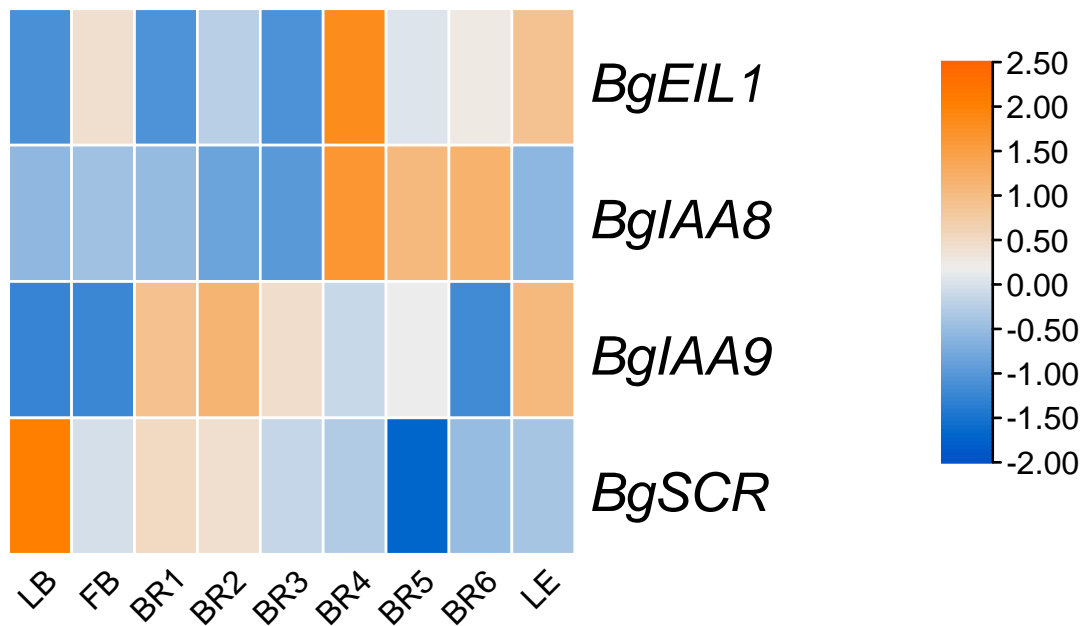

Supplement: Supplementary file 3 — Additional file 3: Fig. S2. Differential gene expression heatmap [file 12870_2024_5246_MOESM3_ESM.pdf]

A

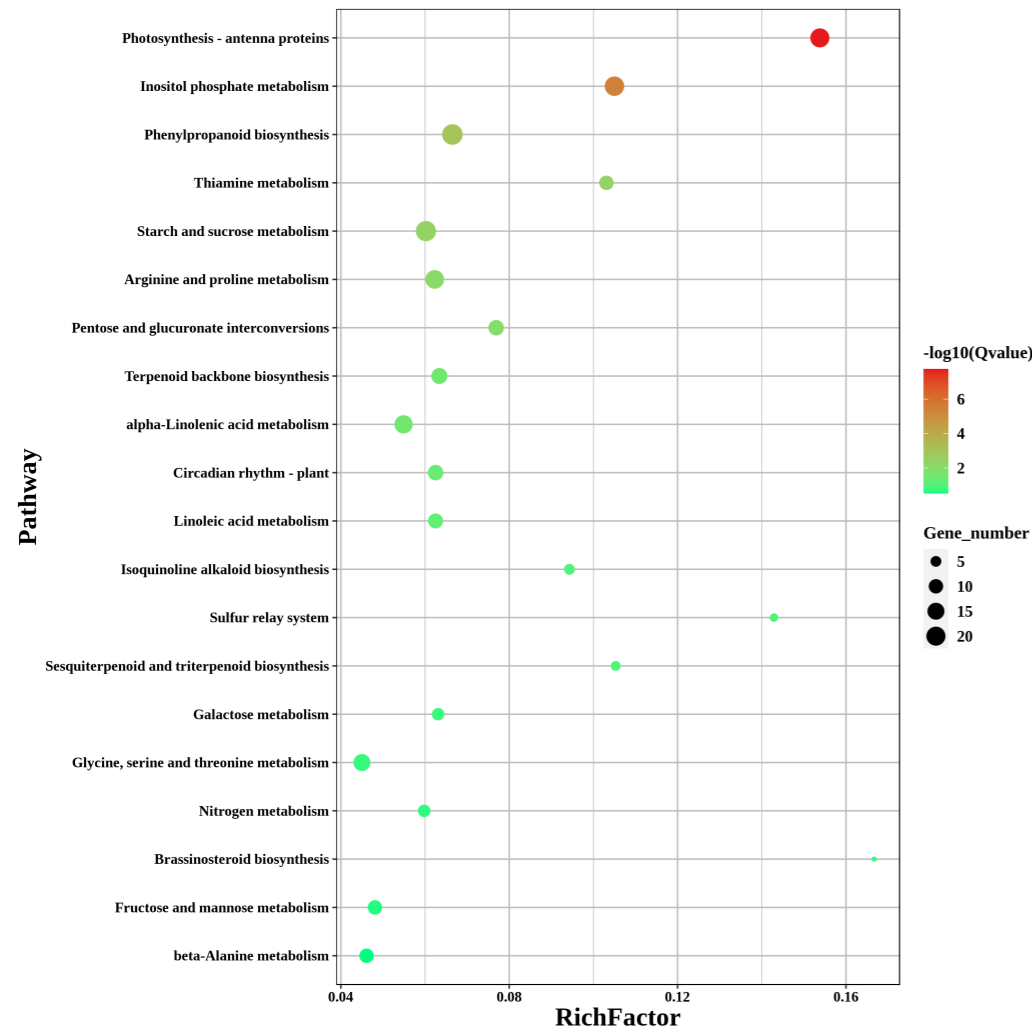

B

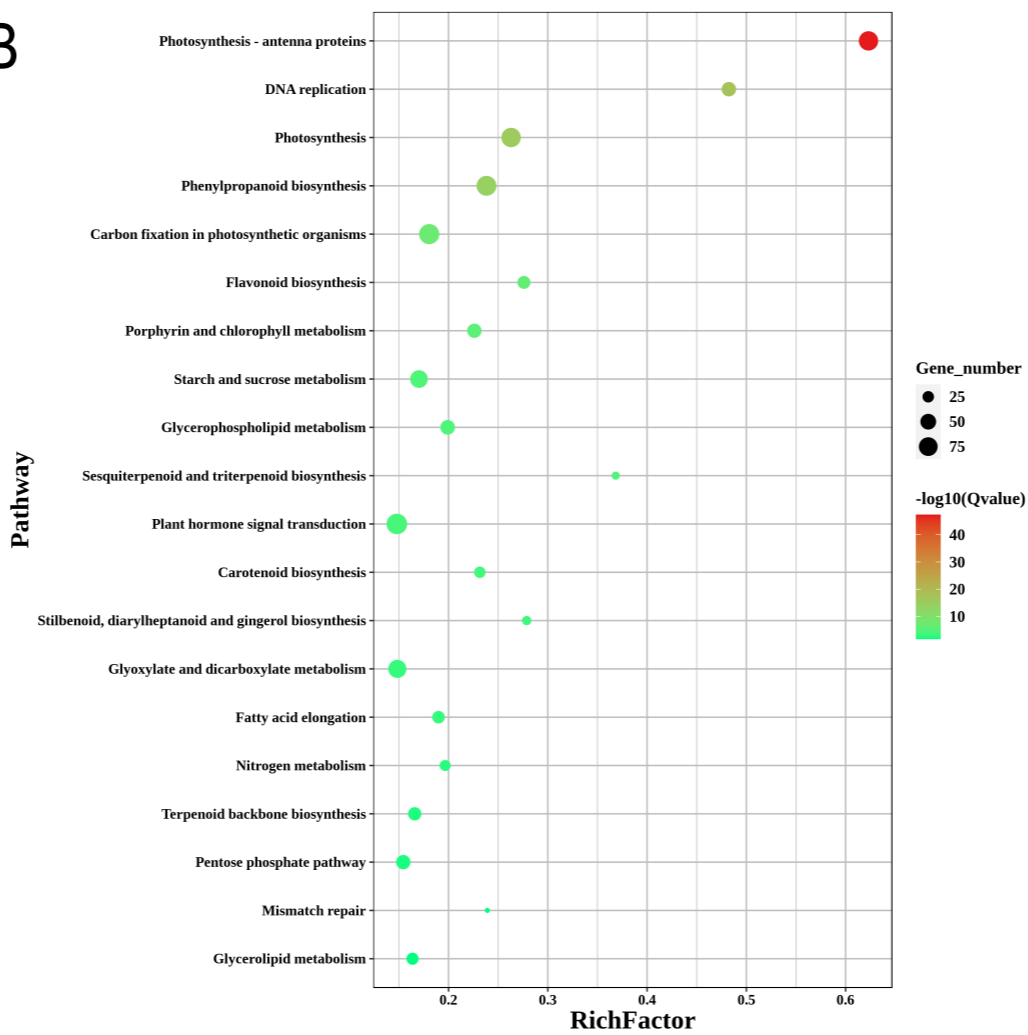

C

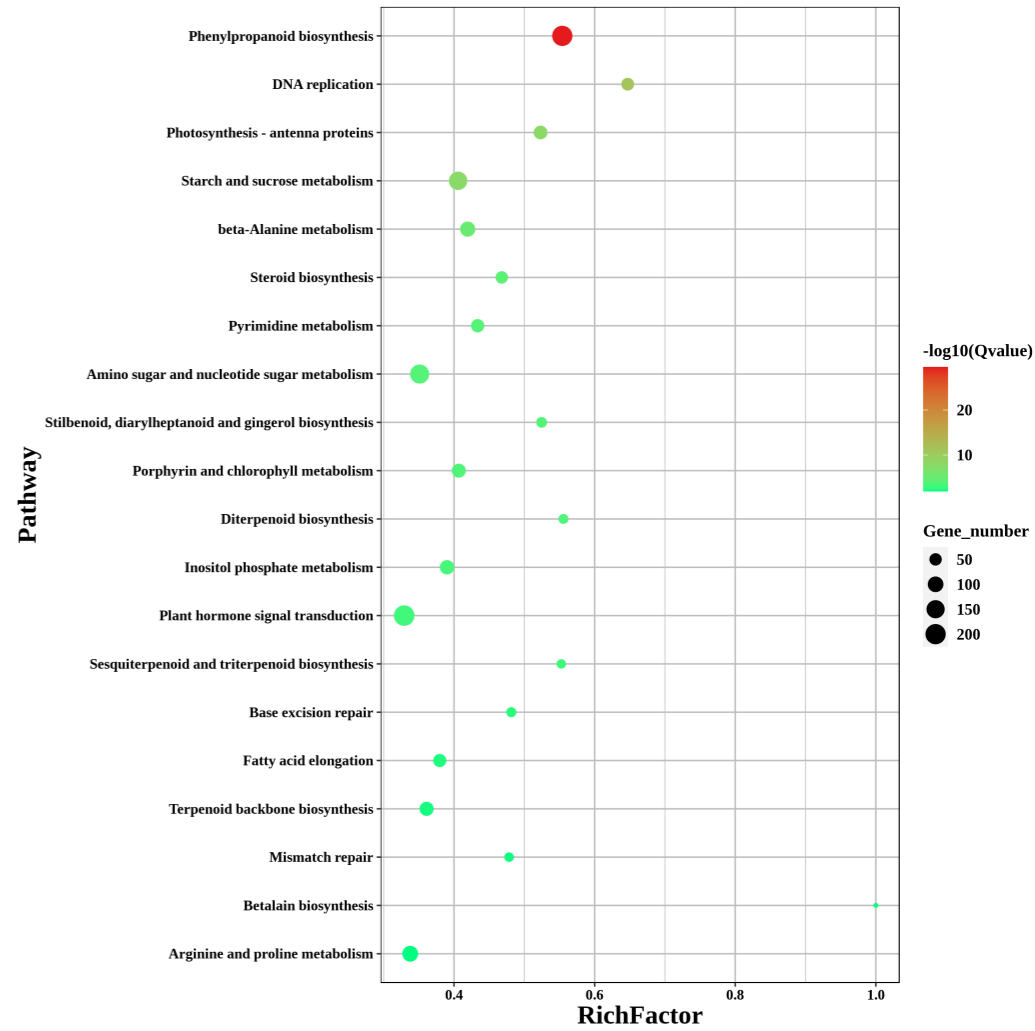

Supplement: Supplementary file 4 — Additional file 4: Fig. S3. Significantly enriched KEGG metabolic pathways at different developmental stages are represented by bubble plots. A FB-VS-BR1. B BR2-VS-BR3. CBR1-VS-BR5 [file 12870_2024_5246_MOESM4_ESM.pdf]

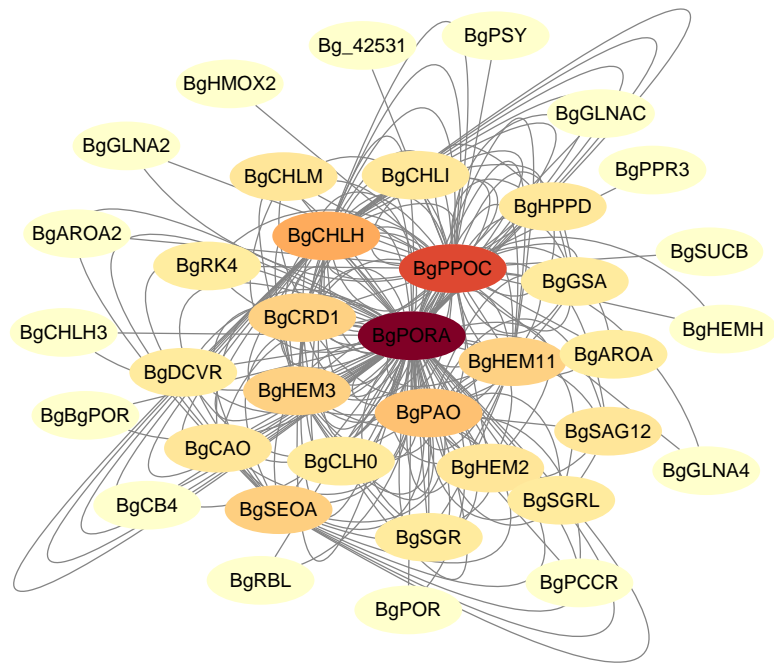

Supplement: Supplementary file 5 — Additional file 5: Fig. S4. Differential protein network interaction map. [file 12870_2024_5246_MOESM5_ESM.pdf]

## Cluster Dendrogram

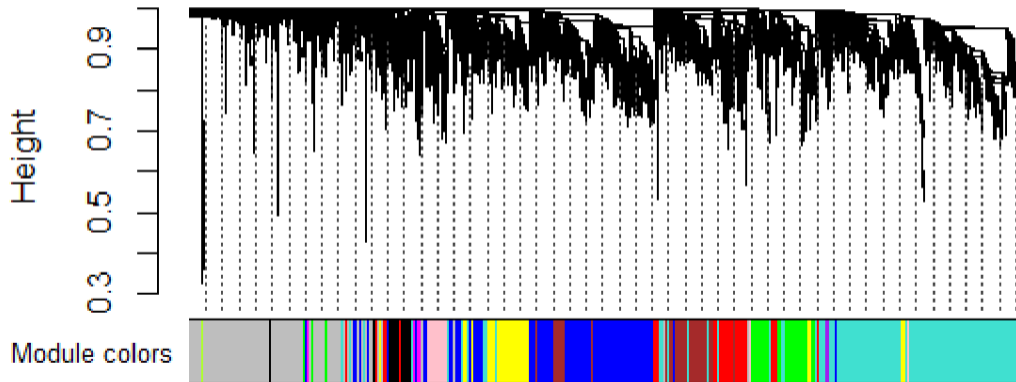

Supplement: Supplementary file 6 — Additional file 6: Fig. S5. Gene module partition map. [file 12870_2024_5246_MOESM6_ESM.pdf]

blue

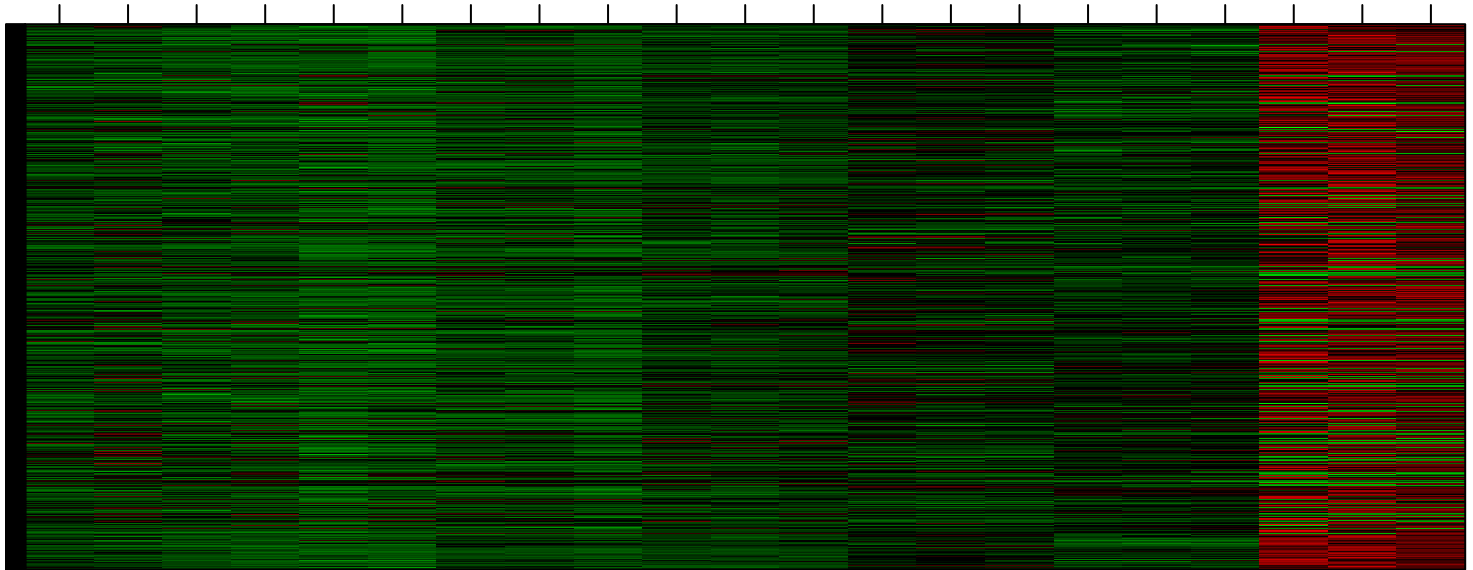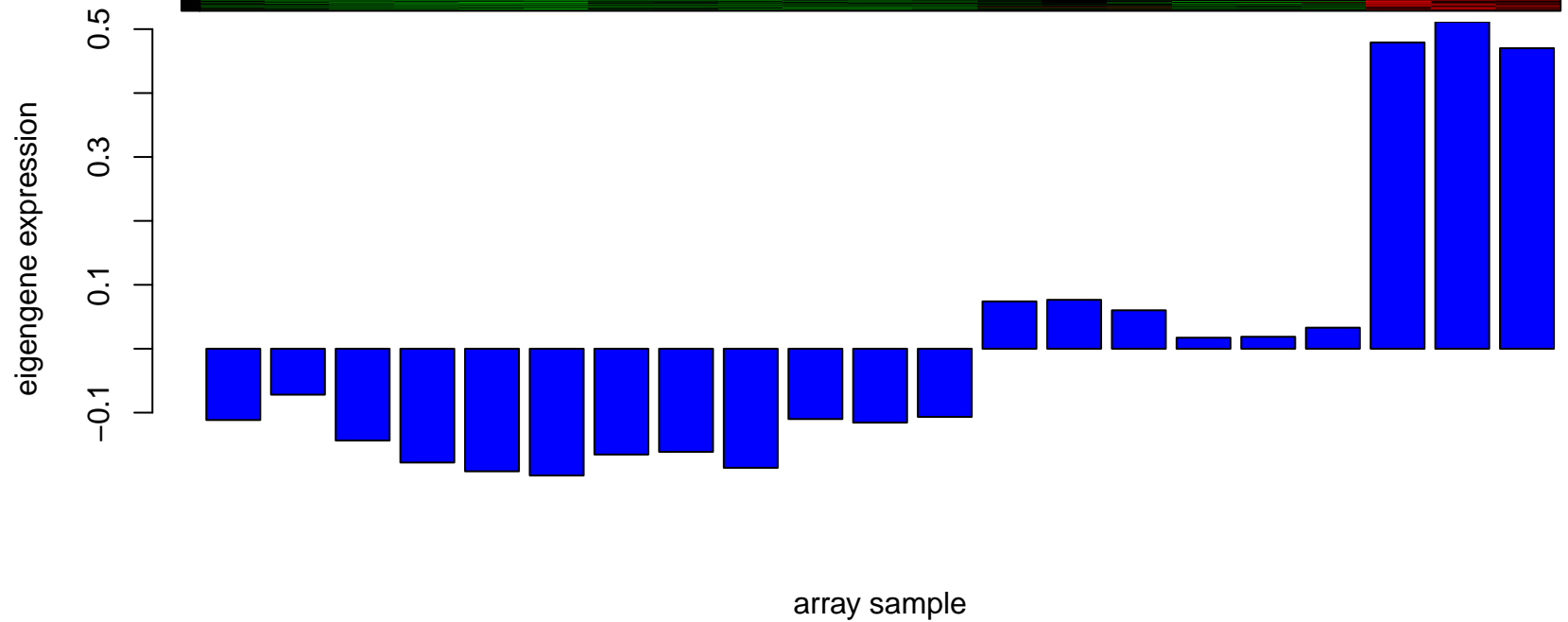

Supplement: Supplementary file 7 — Additional file 7: Fig. S6. Heatmap of gene expression patterns and bar plot of the eigengene expression levels in the blue module. [file 12870_2024_5246_MOESM7_ESM.pdf]

**brown**

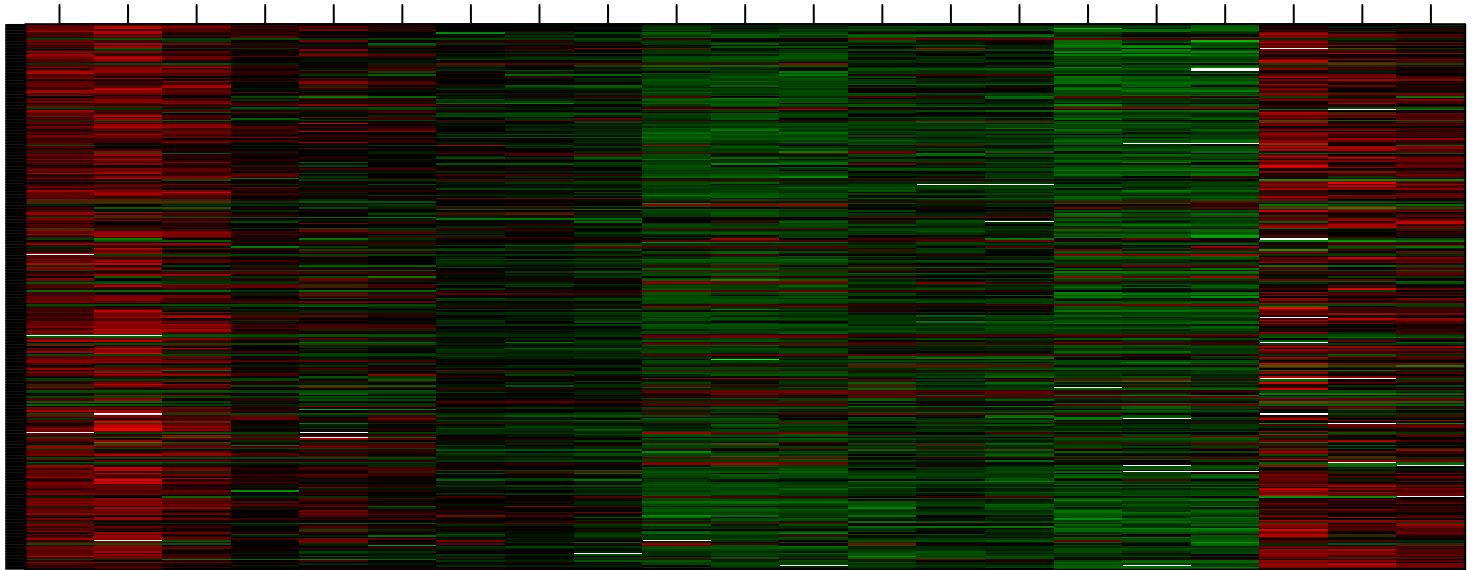

eigengene expression

-0.2  
0.0  
0.1  
0.2  
0.3

array sample

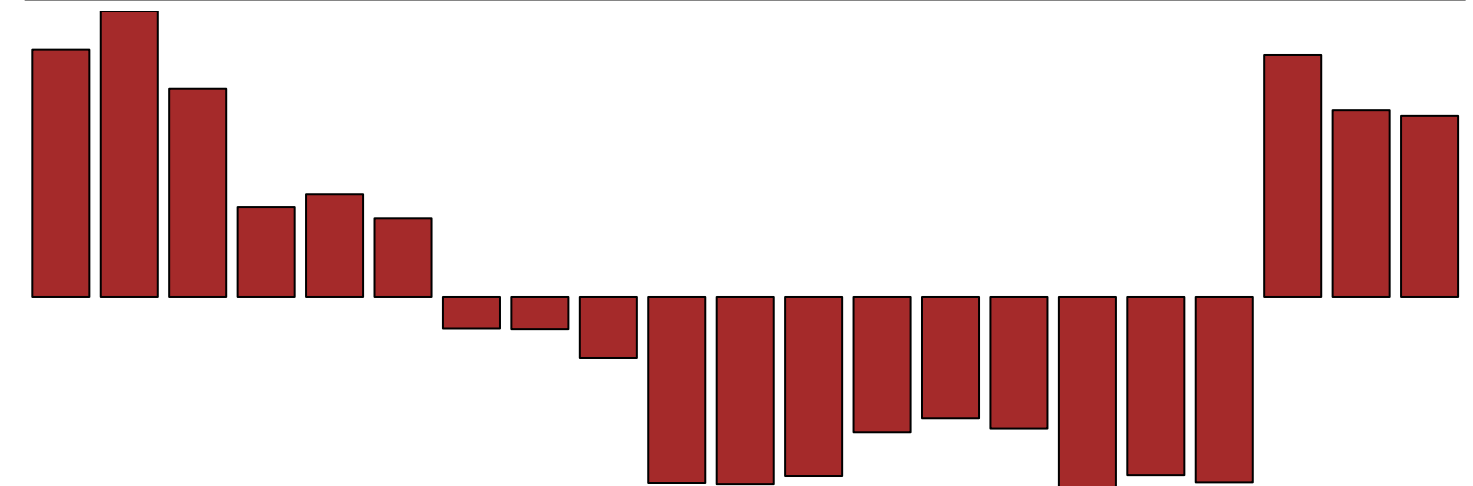

Supplement: Supplementary file 8 — Additional file 8: Fig. S7. Heatmap of gene expression patterns and bar plot of the eigengene expression levels in the brown module. [file 12870_2024_5246_MOESM8_ESM.pdf]

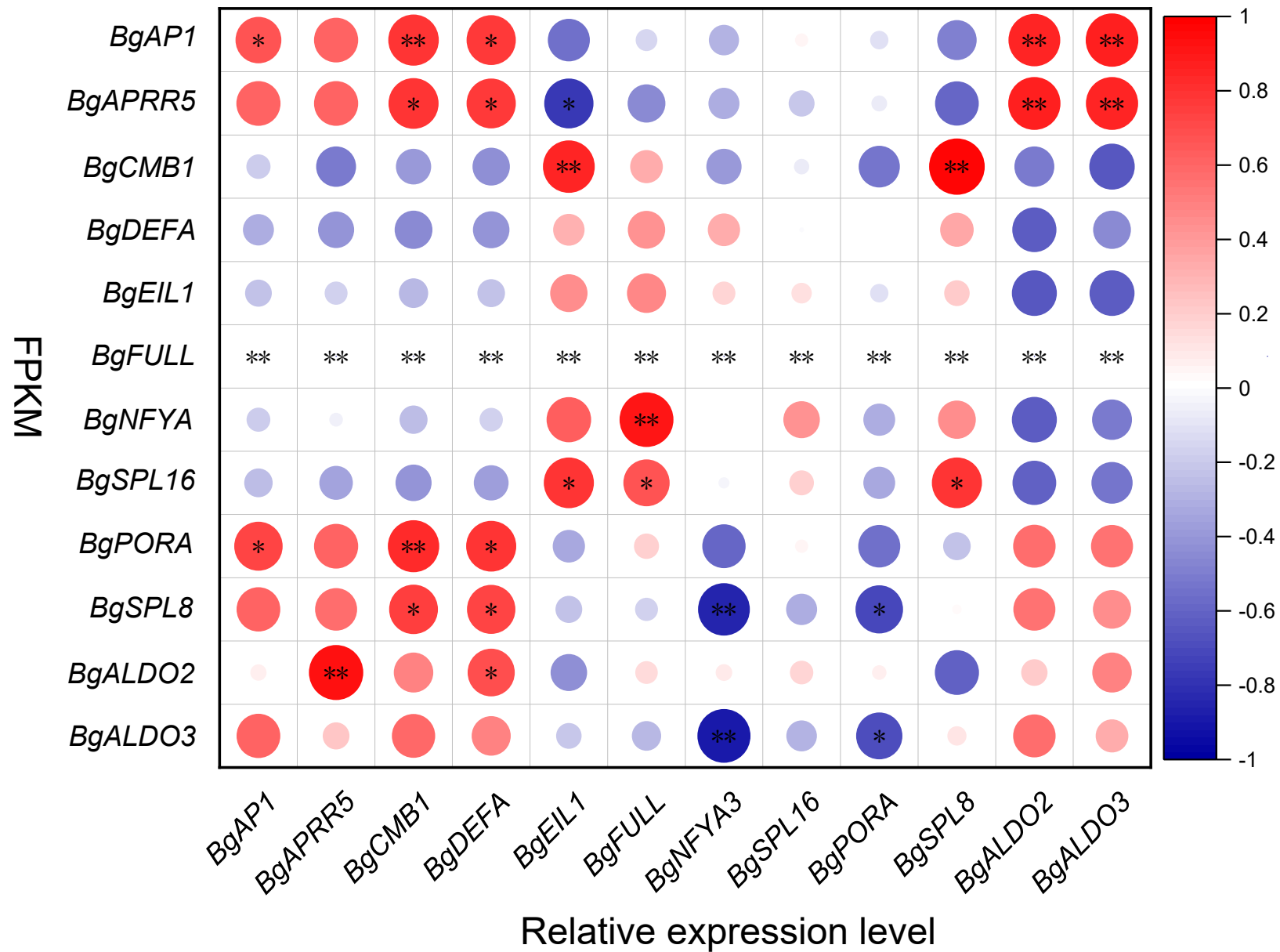

Supplement: Supplementary file 9 — Additional file 9: Fig. S8. Correlation analysis of gene FPKM values with RT‒qPCR [file 12870_2024_5246_MOESM9_ESM.pdf]

**Scale independence**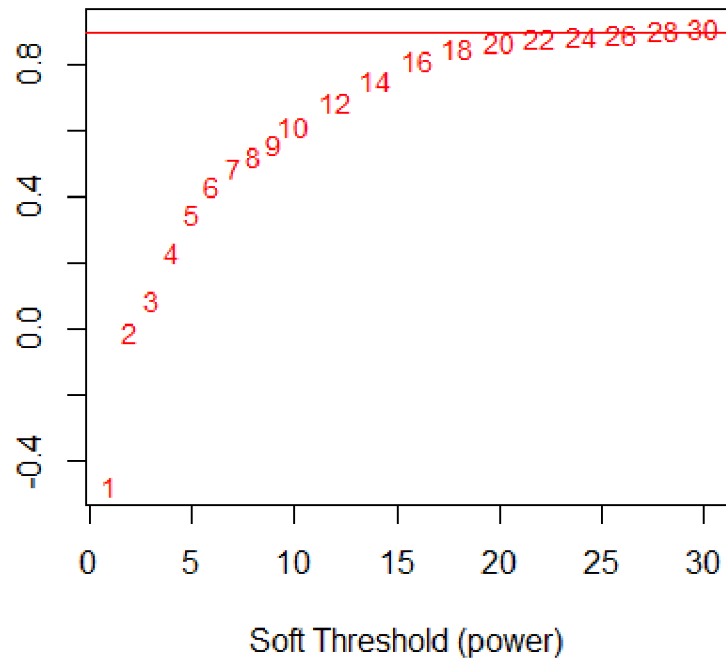**Mean connectivity**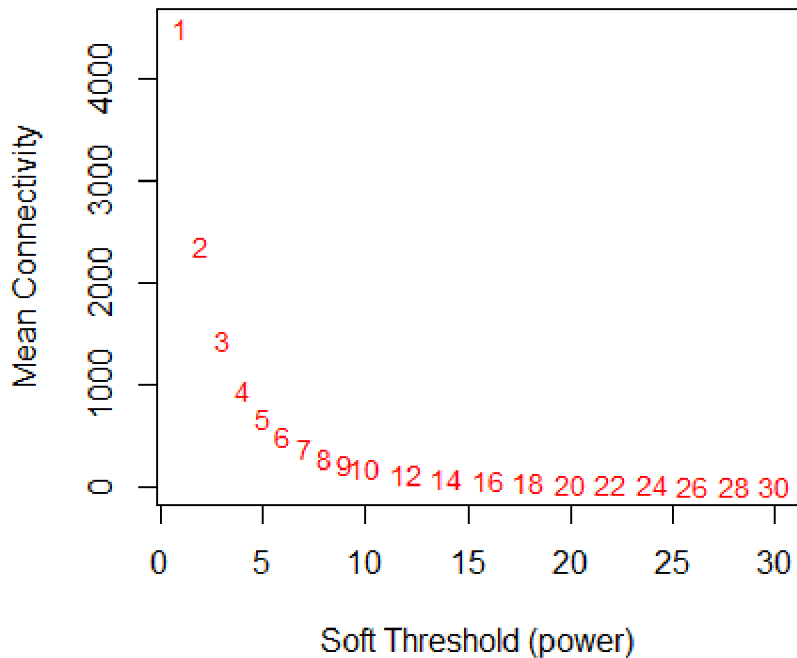

Supplement: Supplementary file 10 — Additional file 10: Fig. S9. Gene scale independence and average connectivity of different powers under the assumption of scaleless networks. [file 12870_2024_5246_MOESM10_ESM.pdf]
